# Transcriptional co-activators: emerging roles in signaling pathways and potential therapeutic targets for diseases

**DOI:** 10.1038/s41392-023-01651-w

**Published:** 2023-11-13

**Authors:** Priyanka Dey Talukdar, Urmi Chatterji

**Affiliations:** Cancer Research Laboratory, Department of Zoology, University of Calcutta, 35 Ballygunge Circular Road, Kolkata, 700019 West Bengal India

**Keywords:** Drug development, Drug development

## Abstract

Specific cell states in metazoans are established by the symphony of gene expression programs that necessitate intricate synergic interactions between transcription factors and the co-activators. Deregulation of these regulatory molecules is associated with cell state transitions, which in turn is accountable for diverse maladies, including developmental disorders, metabolic disorders, and most significantly, cancer. A decade back most transcription factors, the key enablers of disease development, were historically viewed as ‘undruggable’; however, in the intervening years, a wealth of literature validated that they can be targeted indirectly through transcriptional co-activators, their confederates in various physiological and molecular processes. These co-activators, along with transcription factors, have the ability to initiate and modulate transcription of diverse genes necessary for normal physiological functions, whereby, deregulation of such interactions may foster tissue-specific disease phenotype. Hence, it is essential to analyze how these co-activators modulate specific multilateral processes in coordination with other factors. The proposed review attempts to elaborate an in-depth account of the transcription co-activators, their involvement in transcription regulation, and context-specific contributions to pathophysiological conditions. This review also addresses an issue that has not been dealt with in a comprehensive manner and hopes to direct attention towards future research that will encompass patient-friendly therapeutic strategies, where drugs targeting co-activators will have enhanced benefits and reduced side effects. Additional insights into currently available therapeutic interventions and the associated constraints will eventually reveal multitudes of advanced therapeutic targets aiming for disease amelioration and good patient prognosis.

## Introduction

Transcription factors are the principal drivers of multiple diseases.^1^ Numerous studies have highlighted that targeting transcription factors can be exceedingly beneficial in disease diagnosis as well as prognosis.^2,3^ However, most transcription factors are notoriously ‘undruggable’ due to an intrinsic disorder in their structure owing to convex DNA binding interface and flatter protein binding interface, rendering difficulties in targeting their functional associations with DNA or proteins.^4,5^

In principle, transcription of a particular gene can be regulated by modulation of the activity of any component that affects this process.^1^ Transcription factors, in association with transcriptional co-regulators, form multiprotein complexes to translate cellular signals, thereby facilitating transcription of different genes.^6^ The structurally and functionally diverse co-regulators can activate or repress transcription in a cell state-specific manner.^7^ Current advances in research have suggested that co-regulators not only work as transcriptional effectors, but also as delicate metabolic sensors that perceive discrete changes in nutrient and metabolite availability and reproduce transcriptional responses.^8,9^ The co-regulators have been perennially classified into two types, transcriptional co-activators and co-repressors.^10,11^ Amongst them, the co-activators possess the potential to bind transcription factors anchored to DNA in association with catalytic multiprotein complexes and regulate certain epigenetic modifications such as acetylation,^12^ demethylation,^13,14^ allowing effective transcription to take place.^15^ Co-repressors, on the other hand, dock to the transcriptional complexome, and generally mediate deacetylation,^16^ methylation,^17^ thereby, suppressing the transcription of its target genes.

The process of transcription encompasses intricately regulated combinatorial effects of transcription co-activators and co-repressors, as well as time-dependent flexibility, to translate cellular signals maintaining homeostasis.^18^ Even a modest change in either of these factors can disrupt the equilibrium, subsequently inducing series of malevolent traits in the cells and ultimately leading to various disease conditions, including cancer.^19,20^ Researchers have shown that the upregulation of disease-promoting transcription factors is one of the major impulsions of disease progression, propelled by deregulated transcriptional programs involving diverse interweaved actions of transcriptional co-activators.^6,21^ Hence, this transcriptional addiction offers us an alternate art-of-war that can be adopted to reduce the function of the disease-associated transcription factors.

In this review, we begin with an abridgement of co-activator involvement in transcriptional circuitry, followed by the regulation of their activity, expression, and multilateral contributions, in several pathophysiological conditions including developmental disorders, metabolic disorders, with special emphasis on cancer. This accumulated knowledge will enlighten us with recent advances in comprehending the control of gene expression, thereby, rendering novel and attractive opportunities to develop new therapeutic strategies, consequentially targeting the core transcription machinery to curtail disease progression.

## A historical perspective of transcriptional co-activators

**Fig. 1:**
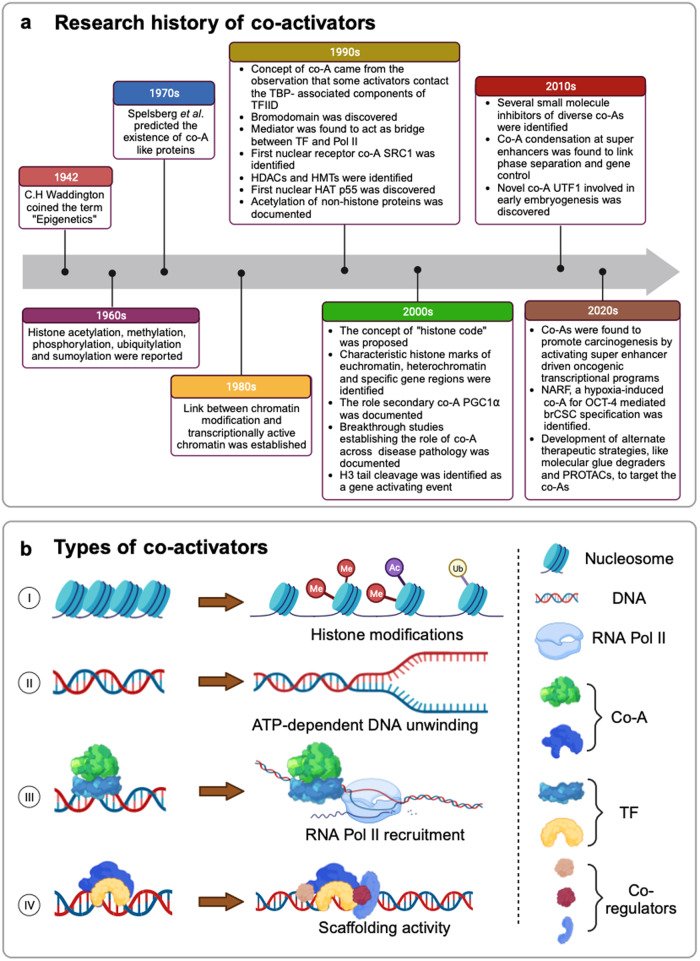
Transcriptional co-activators: history and classification based on mechanism of action. **a** Historical timeline of key events in significant developments of co-activators. **b** Transcriptional co-activators employ diverse mechanistic approaches to augment transcription of target genes. (I) The first class of transcriptional co-activators comprise the proteins that induce posttranslational changes like histone acetylation, methylation and ubiquitination to facilitate euchromatinization and accelerated transcription. (II) The second class facilitates transcription through its ATP-dependent motor activities that induce DNA unwinding activities. (III) This class of co-activators promotes transcription augmentation by enabling the recruitment of RNA polymerase II on the transcriptional machinery. (IV) The final class consists of the secondary co-activators that enhance transcription by serving as scaffolds for the recruitment of other co-regulators. Co-A Co-activator, TBP TATA-box binding protein, Pol polymerase, TF transcription factor, SRC-1 steroid receptor co-activator 1, HDAC histone deacetylase, HMT histone methyltransferase, HAT histone acetyltransferase, UTF1 undifferentiated embryonic cell transcription factor 1. This figure was created using BioRender (https://biorender.com/)

Co-activators, the essential components of cellular functioning, are known to modulate development, cell differentiation, maintenance of stem cells, aging, and their active involvement was recorded in developmental defects, metabolic disorders, and cancer.^22,23^ In the year 1942, Conrad H. Waddington coined the term “epigenetics” to describe the new branch of biology, which describes the regulation of gene transcription and genomic stability without involving alterations in the DNA sequence.^24^ Later, in the 1990s, studies were designed to elucidate the functional roles of the coactivators, initially in yeast.^25^ However, a few years later, the existence of co-factors, the principal epigenetic regulators, was first connoted by transcriptional squelching between estrogen and progesterone receptors.^26^ Since the discovery of SRC-1 (steroid receptor coactivator-1), vast increase in the understanding of the transcriptional control mechanisms of the co-activators have taken place. Numerous co-activators have been isolated, their biochemical properties and molecular mechanisms have been critically evaluated.^27^ Several, non-enzymatic cofactors like TAFs, mediators, and numerous enzymatic cofactors like the histone-modifying cofactors (histone deacetylase, histone acetyltransferase, histone methyltransferase, histone demethylase) and ATP-dependent chromatin-remodeling cofactors (SWI/SNF, ISWI, Mi-2/NuRD, and INO80/SWR1 families) have been discovered since.^28^ Deciphering the functional role of these co-activators has significantly enhanced our understanding of transcriptional co-activator biology.^29^ Based on the significant influence of the co-activators in transcriptional regulation,^6^ more co-activators and their mode of action are yet to be discovered, which will not only foster a better understanding of transcriptional regulation but will also potentiate the development of therapeutic targets across diverse pathological conditions. Timeline of notable findings are illustrated in Fig. 1a.

## Mechanism of transcriptional co-activation

Cell-specific transcription activation is largely regulated by functional interplay between transcriptional co-activators and transcription factors.^6,30^ Therefore, understanding the mechanisms of appropriate co-activator recruitment to facilitate effective transcription is of paramount importance. There are quite a few reports on co-activator recruitment at different stages of transcription.^31–34^ However, the complexity of the mechanism involving co-activators is beginning to be understood. Based on accumulated evidences, different stages of transcription activation and the cross-talk with co-activators during the process, have been summarized.

### Orchestration of co-activators and the transcriptional machinery: symphony of transcription

Co-activators are recruited sequentially during eukaryotic transcription. Removal of repressor complexes marks the initiation of transcription activation.^31^ This is followed by recruitment of DNA-binding transcriptional activators to specific DNA sequences termed as transcription factor binding sites (TFBSs), located in the promoter or enhancer regions of the target gene.^35^ Immediately after recruitment of the activator, the large conformationally flexible mediator complex, which functions as transcriptional co-activator, is recruited to the promoter.^36^ Mediator recruitment eventually promotes docking of chromatin remodeling transcription co-activators. One such family of co-activators is SWI/SNF (SWItch/Sucrose Non-Fermentable), that physically interacts with the mediator to establish nucleosome-depleted regions through nucleosome clearing.^37,38^ SWI/SNF binds to nucleosomal DNA with its translocase domain which is composed of torsion domain and tracking domain. The torsion domain, upon ATP hydrolysis, leads to directional DNA translocation, destroying histone-DNA contacts and creating a transient DNA loop. The DNA loop then propagates around the nucleosome and resolves on the exit site of the nucleosome, inducing nucleosomal repositioning.^39^ After the removal of the nucleosomes from the promoter, the open chromatin state facilitates general transcription factor recruitment, preinitiation complex formation, and RNA polymerase II (RNA Pol II) binding.^40^ The co-activator-mediated nucleosome remodeling is discussed in succeeding sections of this review.

At the next step of transcriptional activation, the general transcription factors (GTFs) are recruited at the promoter.^41,42^ In concordance with the conventional wisdom, the first GTF that is recruited to the promoter DNA is TFIID. Most human promoter DNA contains at least one of the TFIID binding sites: a TATA box sequence upstream of the transcription start site, the initiator element at the transcription start site and the downstream promoter element. The interaction is mediated by TFIID subunits: the TATA-binding protein (TBP) and the TATA-binding protein associated factors (TAFs).^43^ Numerous studies have documented that TAFs function as co-activators and facilitate the interaction between general transcription machinery and the activators.^44^ The GTFs TFIIA and TFIIB are subsequently recruited leading to stable interaction between TBP and the promoter. RNA Pol II is then recruited to the pre-initiation complex probably in association with TFIIF. TFIIE and TFIIH are finally recruited to facilitate DNA melting and formation of transcriptionally competent pre-initiation complex (PIC).^45^ Contrary to the accepted perception, the amino terminus of the mediator subunit MED26 directly interacts with the TFIID subunit TAF7, transforming TFIID to an active structural and functional state.^46^ The mediator complex interacts extensively with Pol II stalk, dock domain and CTD (C-terminal domain) thereby, facilitating the incorporation of Pol II, creating an entire PIC (pre-initiation complex) structure.^47^ TFIIA and TBIIB bind to opposite sides of TBP (TFIID subunit). TBIIB, TFIIE, and TFIIF then directly bind to RNA Pol II.^47^ Owing to its large size, TFIIH interacts simultaneously with TFIIE at the base of the Pol II stalk and position X-box binding protein (XPB) on the DNA. TFIIB linker helix aligns with TFIIF arm at the promoter melting start site, probably facilitating the separation of the DNA strands. The clamp domain starts to swing down during strand separation, prompting the TFIIF arm domain to come closer to the TFIIB B-linker and Pol II rudder, thereby forming a physical barrier for DNA re-annealing. XBP acts as a DNA translocase and inserts the melted single-stranded DNA into the Pol II active site, consequently establishing the open-promoter state of Pol II, which is ready for RNA synthesis.^48^ Eventually, Pol II dissociates from the promoter once the newly synthesized transcript is about 30 nucleotides. Serine 5 on the Pol II CTD is phosphorylated by TFIIH, leading to the recruitment of capping enzyme (CE).^49^ The transcript further undergoes 5’ capping, which protects it from exonuclease-mediated degradation.^42^ This stage is known as early transcript elongation stage. Eukaryotic inactive genes halt at this stage and this process is denoted as Pol II pausing.^50^

Transition from initiation to elongation requires the dissociation of mediator complexes from the promoter. Conceivably, acetylation of lysine 16 residue on histone 4 facilitates mediator complex dissociation from the promoters after completion of its major task in transcription initiation.^51^ For productive elongation to take place, additional modifications at the serine 2 in the CTD (C-tail domain) of the RNA Pol II is required.^52^ The eukaryotic transcription co-activator complex SAGA (Spt-Ada-Gcn5 acetyltransferase) is involved in this process. SAGA has four functionally independent modules: histone acetyl transferase (HAT) module, deubiquitinating module (DUBm), transcription factor (TF) binding module and TBP module. DUBm mediates deubiquitination of H2B which in turn facilitates the recruitment of Ctk1. Ctk1-mediated phosphorylation of Ser2 of RNAPII CTD allows the release of paused Pol II, thereby facilitating elongation of mRNA transcripts.^53^ Subsequently, cleavage of the new transcript and template independent polyadenylation at 3’ end marks transcription termination.^54^

The mRNA export pathway and co-transcriptional mRNP surveillance is regulated by the Sgf73 (a component of SAGA HAT module).^55^ Sgf73 interacts with Sem1p, which is a proteasomal subunit of Sac3p-Thp1p mRNA export complex TREX-2.^56,57^ This interaction induces the separation of the deubiquitylation module from the SAGA complex. The separation facilitates localization of mRNA export factor Mex67-Mtr2 and TREX-2 to the transcriptional machinery, consequently leading to mRNA export.^58^ Another component of the DUB module, Sus1p, which is also associated with TREX-2, is responsible for targeting of genes to the NPCs (nuclear pore complexes).^59^

### Modulation of chromatin looping by the co-activators

Prior to the emergence of topological associating domains (TADs), the precise control of transcriptional activation relies on the interaction between remote cis-regulatory modules (e.g., enhancers, and the promoter. The formation of chromatin loops before the recruitment of the activators facilitates the communication between the promoters and enhancers.^60^ The transcriptional co-activators have also been reported to play significant role in this process. The transcriptional co-activators YAP and TAZ promote recruitment of the mediator complex at the enhancer, thereby establishing long range chromatin looping and facilitating enhancer-promoter contacts to recruit lineage-specific transcription factors.^61^ The mediator co-activator complex further acts as a bridge to relay information from the enhancers to the promoters. The tail module of mediator complex associates with the enhancer bound transcription factors while the other modules bind to Pol II and PIC at the promoter to dynamically link the promoter and the enhancer.^62^

### Co-activator interaction with transcription factors

Transcription factors can bind to DNA in a sequence-specific manner^63^; however, these principal regulators need assistance from several other factors to regulate chromatin remodeling, DNA unwinding, and RNA polymerase II recruitment, which are necessary for effective transcription to take place. These biochemical activities are the speciality of the transcriptional co-activators, which are multiprotein complexes that dock on the DNA-binding activators.^64^ The sequence-specific transcription factors contain variable and intrinsically disordered transcription activation domains (TADs).^65^ Interaction with the TAD domain of site-specific transcription factors, mediates the positioning of the transcriptional co-activators at promoter regions,^66^ where they induce chromatin remodeling and act as bridges between general transcription machinery and the activator, hence promoting transcription activation.^67^ For example, CBP/p300 histone acetyl transferase interacts directly with C-terminal transactivation domain of E2F transcription factor.^68^

Moreover, the TAD domain of transcription factor exhibit “structural plasticity” which propels an adaptive association with multiple co-regulatory molecules.^69,70^ A study by Marceau et al.^71^ has reported that the transcription factor FOXM1 contains a disordered TAD. When in association with negative regulatory domain (NRD), the TAD domain attains order in its structure. However, dissociation from NRD restores the disordered conformation. The disordered TAD is then capable of binding the transcriptional co-activator CBP.

The conventional model of co-activator mediated gene transcription indicates passive role of the transcription factor, where they are only responsible for localizing the co-activator complexes to the genes.^71^ However, some studies have also reported that the docking of the co-activator on the transcription factor switches on the co-activator activity. A study has reported that CBP and P/CAF when bound to mutant HNF-1α transcription factor do not exhibit HAT activity, indicating that the transcription factors not only recruit the co-activators at the promoter region but also modulate their enzymatic activity.^72^ Another study has demonstrated that the transcriptional co-activator PGC-1α exhibits a quiescent stage when not bound to transcription factor. However, interaction with transcription factors induces a conformational change and promotes the interaction of PGC-1α with SRC-1 and CBP/p300.^73^

### Modulation of chromatin structure by co-activators

Chromatin has been reported to be an instructive DNA scaffold that can respond to intracellular and extracellular cues, and act to regulate the many uses of DNA.^74^ One of the most significant ways to regulate transcription has been to influence chromatin packaging, which determines the availability of DNA elements.^75^ This is achieved by two major type of modifications, covalent histone-modifications and ATP-dependent chromatin remodeling.^76^ Methylation,^17^ acetylation,^12^ ubiquitination,^77^ demethylation^13^ and deubiquitination,^78^ the crucial histone modifications introduced by the co-activators, are principle regulators of chromatin structure and are involved in the manipulation and expression of gene.^79^ On the other hand, ATP-dependent chromatin-remodeling complexes guide gene expression by restructuring the nucleosome.^80^ Complicated integration and synchronization of these modifications not only regulate chromatin structure but recruit the transcriptional machinery and govern target gene expression.^81^

#### Methylation

Methylation of the histone proteins is one of the important phenomena regulating chromatin structure and gene transcription.^17^ Nucleosomes, the fundamental unit of chromatin, is composed of a stretch of DNA wrapped around a protein octamer consisting of two copies each of the four histone proteins: H2A, H2B, H3, and H4.^82^ All of these proteins possess a tail extension, which is targeted for methylation.^82^ Histones can be methylated on two amino acid residues, lysine (K) and arginine (R); however, lysine residues of histone tails are mostly of preference.^83^ Several transcriptional co-activators possess the methyl transferase activity and are known to modulate the histone architecture to promote transcription.^84^ Histone methylation has been found to be associated with both compact and relaxed chromatin structure, depending on the methylation sites.^85^ For instance, higher H3K4me1/2/3, H3K36me3 and H3K79me1/2/3 helps in euchromatinization; on the other hand, heterochromatinization is characterized by higher levels of H3K9me2/3, H3K27me2/3 and H4K20me3.^86,87^ The lysine methyl transferase KMT DOT1L demethylates histone H3 at lysine 79 (H3K79me2) which promotes lineage-specific gene expression to regulate T_H_ cell function.^88^ Another lysine methyl transferase (SET8/KMT5A) is the only mammalian enzyme known to monomethylate histone H4 at lysine 20 (H4K20me1). This particular histone modification plays important roles in DNA damage repair by recruiting signaling proteins like 53BP1 to the site of double-stranded DNA breaks.^89^ In addition, protein arginine N-methyltransferases (PRMTs) asymmetrically dimethylate 3 (R3) residue of H4, potentiating subsequent histone acetylation, and contributes to the maintenance of an active chromatin structure. This suggests that such histone modifications can function as a transcriptional activation mark.^84^ Methylation of core nucleosomal histones can either activate or repress transcription depending on which amino acid residues in the histones are methylated and how many methyl groups are attached. Deregulation of methylation has been found to cause neurodegenerative diseases, metabolic disorders, and cancer.^90–92^

#### Acetylation

Acetylation of histones is highly dynamic and regulated by the opposing action of two family of enzymes, histone acetyltransferases (HATs) and histone deacetylases (HDACs).^12,16^ Transcriptional co-activators utilize acetyl CoA as cofactor and catalyse the transfer of an acetyl group to the ε-amino group of lysine side chains,^93^ thereby neutralizing the positive charge of lysine that weakens the non-covalent electrostatic interactions between histones and negatively charged phosphate groups of DNA.^94^ As a consequence, the condensed chromatin state is transformed into a more relaxed euchromatin to enable greater accessibility of DNA and promotes transcription of related genes.^95^ Each of these molecules can modify multiple sites within the histone N-terminal tails, which in turn dictates the subsequent histone modifications.^79^

A study demonstrated that the transcriptional co-activator p300 facilitates acetylation of H3K122 in the globular domain via interacting with BRG1 (Brahma-related gene 1). This in turn destabilizes histone-DNA binding and assists transcription.^96^ Hassan et al.^97^ reported that histone acetylation by SAGA complex stabilizes binding of SWI/SNF binding to the nucleosome to mediate ATP-dependent chromatin remodeling. SWI/SNF has also been shown to interact with p300 and regulate H3K27 acetylation to enhance transcription.^98^ In addition to the histone tails there are also other sites of acetylation present within the globular histone core, such as acetylation at H3K56 in humans by hGCN5.^99^ Apart from hGCN5, the transcriptional co-activator p300 is also reported to be associated with H3K56ac. Strikingly, knockdown of p300 induces loss of H3K56ac and increase in DNA damage, establishing a prominent role of the transcriptional coactivator-mediated histone acetylation in nucleosome remodeling.^100^

#### Ubiquitination

In addition to the above, ubiquitination is another type of reversible histone modification. Ubiquitination is a process that ligates ubiquitin, a 76 amino acid protein, on lysine residues of histones, by covalent interaction through an isopeptide bond between its C-terminal glycine and the ϵ-amino group of a lysine residue.^101^ The mechanisms by which histone ubiquitination affect transcription are multiple. Histone ubiquitination can alter higher-order chromatin folding and provide greater access of the underlying DNA, which may function as a signal for the recruitment of transcription regulatory molecules.^102^ It is also possible for ubiquitination to act as an integrator of different post transcriptional modifications on histones.^103^

Mono-ubiquitination of histone H2B at lysine 123 in *Saccharomyces cerevisiae* or at lysine 120 in mammals is necessary for maintaining stable altered nucleosome state for transcription.^104,105^ Functional human homologs of the yeast BRE1 E3 ubiquitin ligase are the transcriptional co-activators RNF20 and RNF40. The co-activator molecule RNF20 enhances the global level of ubiquitylation at lysine 120 of histone H2B, thereby promoting activator-dependent transcription.^106^ A study by Krajewski et al.^107^ has shown that ubiquitination of H2BK34, which is surrounded by two coils of DNA superhelix, directly influences nucleosome conformation via steric hindrances. Petty et al.^108^ have demonstrated that H2B is ubiquitylated by co-activators RAD6 and BRE1 which is associated with gene activation in yeast and mammals. Histone H3B monoubiquitylation has also emerged as a new regulator for heterochromatinization in metazoans.^109^ The study indicates that co-activator-mediated ubiquitination is definitively associated with gene activation.

#### Demethylation

Histone demethylation is dynamically regulated by the activity of histone demethylases that are categorized into two families: KDM1 family/Lysine specific demethylase 1 (LSD1) and Jumonji C (JmJC) domain-containing histone demethylases.^110^ KDM1 family of nuclear amine oxidase homolog removes mono- and di-methylated lysine of H3 at lysine 4 or 9 in a cell-state specific context. In contrast, the JmJC domain-containing demethylases, belonging to the 2-oxoglutarate-dependent dioxygenases, are capable of removal of trimethylations. Wissmann et al.^111^ reported that KDM1A demethylates H3K9me1 and H3K9me2 when complexed with androgen receptor, leading to transcription activation, and Cloos et al.^112^ reiterated that KDM4C, in particular, increases euchromatin available for transcription. Several in vitro differentiation studies have established the necessity for the KDM6 H3K27me2/3 demethylases, KDM6A/UTX and KDM6B/JMJD3, in overcoming the repressive chromatin state and initiate normal transcription.^113–116^ Another fascinating study by Tsai et al.^117^ showed the interaction between lncRNA HOTAIR and KDM1A/CoREST complex, which recruits the demethylase complexes to the target site, creates a repressed chromatin state. However, histone demethylases have been shown to have enigmatic biological interactions and current studies indicate contradictory function in transcription activation. Further studies are imperative to establish their role in promoting transcription.

#### Deubiquitination

The process of deubiquitination involves the removal of ubiquitin molecule from the target proteins and dissolution of ubiquitin complexes.^17^ BAP1, a ubiquitin C-terminal hydrolase (UCH) domain-containing protein, promote gene expression by catalyzing removal of monoubiquitination on lysine 119 of histone H2A (H2AK119ub1) through a multiprotein complex.^118^ Deubiquitination module of the SAGA complex, that comprises Usp22, Eny2, and Atxn7, deubiquitinates H2BK120ub following DNA damage, which is critical for class switch recombination.^119^ USP22, a well-known role co-activator of VEGF-A, specifically plays a crucial role by reversing the ubiquitination by ubiquitylating enzymes. It serves as ubiquitin hydrolase and catalyzes the deubiquitination of H2A and H2B, thereby counteracting heterochromatin silencing and promoting gene transcription.^120^ Another study by Ducker et al.^121^ has reported that USP17 induces deubiquitination of the transcription factor ELK-1 at lysine 35, consequently upregulating its transcription. However, the mechanism underlying deubiquitination-mediated transcription activation is yet to be defined with clarity.

#### ATP-dependent chromatin remodeling

Chromatin remodeling involves changing the histone-DNA interactions by disrupting, assembling or nucleosome sliding.^122^ This process is carried out by a family of enzymes with ATPase and helicase ancestry, the chromatin remodeling enzyme complexes. These remodeling enzymes induce partial dismantling of nucleosomes, liberating segments of DNA and rendering them accessible to the interacting proteins.^123^

Till date, 4 classes of these remodeling enzymes have been identified: SWI/SNF, ISWI, CHD, and INO80.^124^ Coincidentally all these enzymes function as transcriptional co-activators. SWI/SNF is one of the first described chromatin remodeler enzymes that is recruited to the promoter at the same time as the transcription activators. Upon ATP hydrolysis, SWI/SNF carries out a directional DNA translocation, which destroys DNA-histone binding, causing the nucleosome to reposition.^125^ The ISWI family of remodelers regulate DNA accessibility by mobilizing nucleosomes and controlling the length of linker DNA separating nucleosomes by a mechanism that is not very lucid till date.^126^ The evolutionarily conserved INO80 family of ATP-dependent chromatin-remodeling enzymes modify chromatin in a number of ways including nucleosome sliding and exchange of variant histones. INO80, along with SWI/SNF remodelers, promotes nucleosomal clearing of PHO5 gene promoter.^127^ However, information about the role of INO80 in transcriptional co-activation is limited; therefore, conclusive statement about the mechanism of their execution cannot be stated. Biochemical analyses revealed that chromodomain helicase DNA (CHD)-binding proteins affect DNA-histone interactions within the nucleosome in a manner that is distinct from the yeast SWI/SNF complex.^128^ Interestingly, they are often linked with maintenance of pluripotency in embryonic stem cells.^129^

## Types of transcriptional co-activators

Depending on their mechanism of action, transcriptional co-activators can be broadly categorized into four different classes.^30^ The first class of co-activator proteins performs histone modifications, resulting in dispersed structure of chromatin, thereby rendering it accessible to transcription factors.^130^ The second class comprises proteins that possess ATP-dependent DNA chromatin remodeling activity, thus augmenting transcription.^131^ The third class interacts with general transcription apparatus and recruits RNA pol II to promote transcription initiation and elongation.^132^ The fourth class of co-activators, known as the secondary co-activators, interacts with transcription factors and function as scaffolding non-enzymatic proteins to recruit other co-activators containing enzymatic activities on the target gene promoter.^30^ Furthermore, several transcriptional co-activators exhibit the properties of both primary and secondary co-activators in variable contexts^133,134^ (Fig. 1b).

## Overview of various co-activator families

Several families of proteins have been characterized and classified as transcriptional co-activators. Here, we have attempted to elaborate the structural conformation and the cell-specific functions of different co-activator families.

### BET family

The four conserved members of the BET (bromodomain and extraterminal domain) family of proteins in the mammals are BRD2 (also known as FSRG1, RING3, RNF3, FSH, or D6S113E), BRD3 (also known as ORFX or RING3L), BRD4 (also known as MCAP or HUNK1) and BRDT (also known as BRD6, CT9, or SPGF21).^135^ The bromodomain-containing proteins (BRDs) have been recognized to function as epigenetic readers.^136^ Epigenetic readers are a group of specialized docking domain containing proteins that identify and bind to various covalent modifications on histones, non-histone proteins and DNA. BRDs specifically recognize acetylated lysine residues in histone H3 and H4.^137^ For instance, a study has reported that IL1β or TNF-induced acetylation of H4K5Ac, H4K8Ac, and H4K12Ac mediates the recruitment of the BET proteins, BRD3 and BRD4, to the matrix degrading enzyme gene promoter, consequently upregulating their expression in human chondrosarcoma cells.^138^ Histone H3 acetylation, especially at H3K18Ac, facilitates the recruitment of BRD3 and BRD4 to the promoter of CXCL8 gene which encodes interleukin-8 protein. This promotes the expression of IL-8 in airway smooth muscle cells and drives steroid-resistant neutrophilic airway inflammation in asthmatic individuals.^139^

BRD4 can also function as an atypical histone acetyl transferase. However, the mode of acetylation is distinct from other HATs as BRD4 has the property to induce acetylation of histone H3 on Lys residue 122 (H3K122Ac), leading to destabilization of nucleosome structure and chromatin destruction.^140^ The HAT activity of BRD4 has been documented in inflammation-driven airway remodeling.^141^ BETs can also interact with transcription elongation complexes and transcription factors through lysine acetylation-dependent or independent mechanisms.^142^ The positive transcription elongation factor, P-TEFb is a cyclin-dependent kinase comprising CDK9 and other cyclin subunits like cyclin T1.^143^ The BETs are responsible for recruiting CDK9 and cyclin T1 to RNA Pol II.^138^ This interaction mediates the phosphorylation of Ser2 and Ser5 of Pol II C-terminal domains, thus allowing productive elongation.^144^

### SRC family

The steroid receptor co-activators of p160 family consisting of three homologous members SRC-1 (also known as NCOA1), SRC-2 (also known as TIF2, GRIP1 and NCOA2) and SRC-3 (also known as p/CIP, RAC3, AIB1, ACTR, TRAM1, and NCOA3) has been recognized to regulate a plethora of physiological processes. The SRC family of proteins possess three structural domains.^145^ The conserved basic helix-loop-helix-Per/ARNT/Sim (bHLH-PAS) domain, located in the N-terminal, is required for interaction with transcription factors and contains a canonical NLS (nuclear localization signal).^146^ The central region consists of three LXXLL motifs (X is any amino acid). This region mediates interaction with transcription factors and the nuclear receptors. Central region also contains a serine/threonine-rich domain which upon phosphorylation influences the SRC activity.^145^ Two transcriptional activation domains (ADA1 and ADA2) are located in the C-terminal.^147^ The ADA1 activation domain is involved in binding with the transcriptional co-activators CBP/p300. The SRCs exerts their role in chromatin modification through this interaction with CBP/p300.^148^ The ADA2 activation domain binds to the histone methyltransferases CARM1 (co-activator-associated arginine methyltransferase 1) and PRMT1 (protein arginine N-methyltransferase 1) to facilitate transcription activation.^149^

SRC-1 co-activators are involved in regulating carbohydrate metabolism. SRC-1 has been reported to initiate gluconeogenic program through transactivating pyruvate carboxylase by modulating the activity of C/EBPα.^150^ SRC-1 has also been reported to control insulin signaling by modulating the expression of insulin receptor substrate 1 (IRS1).^151^ SRC-2 has been determined to be a positive regulator of mammalian circadian rhythm as they function as transcriptional co-activators of the brain and muscle ARNT-Like 1 (BAML1) and circadian locomotor output cycles kaput (CLOCK).^152^ SRC-3 has been widely reported to be amplified in tumors.^153^ SRC-3 modulates the AKT signaling pathway to stimulate prostate and ovarian cancer cell growth and promote glycolysis in urinary bladder cancer, by upregulating the expression of GLUT1 and PGK1 genes via its interaction with HIF1α (hypoxia inducible factor 1α).^154^ Given their role in coordinating energy accretion and utilization in the context of normal physiology and malignancy, the SRC-family of transcriptional co-activators is an emerging area of concern.

### KMT family

The lysine methyltransferase (KMT) family of transcriptional co-activators methylates histones and consists of 23 different SET proteins and one 7βS protein (a total of 24 different enzymes).^155^ The methyl transferases contain a SET domain, and flanking the SET domains are a pre-SET domain and a post-SET domain. Pre-SET domain stabilizes the structure by forming triangular zinc clusters using cysteine residues. The SET domain contains a catalytic core composed of β-strands.^156^ The lysine residues in the histone tail of the substrate and the S-adenosyl methionine (SAM) are bound and oriented into the SET domain to initiate methylation.^157^ This promotes S_N_2 nucleophilic attack of the ε-amine that leads to transfer of methyl group from SAM to lysine, thereby introducing monomethyl-lysine.^158^ Following an initial round of methylation, the monomethyl or dimethyl lysine residues are oriented for subsequent methylation events.^159^

Several studies have reported the role of KMT family of proteins in transcriptional regulation. KMT2C/D COMPASS complex of methyl transferases and their interacting partners promote active euchromatic conformations by modification of histone-3 tail residues.^160^ Cyclin D1-mediated recruitment of lysine methyltransferase (KMT) G9a/EHMT2 induces H3K9me2 that promotes positioning of chromosomes by facilitating the interaction between nuclear lamina (NL) and the lamina-associated domains (LAD).^161^ However, accumulated evidences indicate that methylation-mediated transcription suppression is also predominant. Tanaka et al.^162^ suggested that SETD8/PR-SET7-mediated mono methylation of histone H4 at lysine 20 leads to repression of p16INK4A and ribosome-associated genes that are associated with senescence. SET7/9-mediated methylation of FoxO3 K270 prevented FoxO3 interaction with its target genes and prevented the transcriptional activation of FoxO3, indicating that the site of methylation regulates diverse biological processes.^163^

### CBP/p300 family

Two paralogous acetyl transferases that have been widely recognized to function as transcriptional co-activators to enhance transcriptional activation are CREB binding protein (CBP) and p300. CBP, also known as KAT3A, is encoded by the CREBBP gene and p300, also known as KAT3B, is encoded by EP300 gene.^164^ Both the paralogous transcriptional co-activators contain highly conserved modular structure that encompasses an acetyltransferase domain, acetyl lysine-binding bromodomain (BD) and diverse structured modules like KIX domain, the cysteine/histidine regions (TAZ1 and TAZ2), the interferon response binding domain (iBID) and the nuclear receptor interaction domain (RID).^165^ According to Shikama et al.^166^ nucleosome assembly protein/template activating protein (NAP/TAF), which functions as histone chaperones, can functionally interact with p300 co-activator proteins. The histone 3 lysine 27 acetylation (H3K27ac) activity at regulatory elements such as enhancers and promoters, that is mediated by the acetyl transferases CBP and p300 is required for cell type-specific gene expression patterns.^167^ At specific regions of the genome in the mouse embryonic stem cells, p300 is responsible for maintaining H3K27ac, according to Martire et al.^168^ p300 interacts with Glut2 promoter and the transcription factor HNF1α to upregulate the expression of Glut2, a major glucose transporter in the hepatocytes and the pancreatic β-cells.^169^ Owing to their diverse gene regulatory role, deregulation of p300/CBP contributes to various pathological conditions.

### CRTC family

The cAMP response element binding protein (CREB) has been documented to function in association with a family of co-activators known as cAMP-regulated transcriptional co-activators (CRTCs).^170^ They are also referred to as transducer of regulated CREB activity (TORC) or mucoepidermoid carcinoma translocated protein (MECT).^171^ CRTCs are highly phosphorylated at basal conditions and are retained in the cytoplasm through interactions with 14-3-3 proteins. Rise in cAMP and calcium level induces calcineurin-mediated dephosphorylation of CRTC that facilitates its release from 14-3-3 complexes.^170,171^ CRTC family of co-activators comprise three members: CRTC1, CRTC2, and CRTC3.^172^ Mutational analyses have also showed that the CRTCs contain distinct functional domains that are responsible for regulating pre-mRNA splicing.^173^ CRTC family of CREB regulated transcription co-activators are involved in cAMP-pathway-mediated melanocyte differentiation. CRTC3 binds to a conserved enhancer of CREB and leads to upregulation of oculocutaneous albinism 2 (OCA2) protein expression, which then promotes melanosome maturation. CREB/CRTC1 pathway further influences the neuronal activity-dependent gene transcription.^174^ CRTC1 upon dephosphorylation due to neuronal activity is translocated to nucleus, where it binds to the transcriptional complexome in CRE/TATA promoters to promote neuronal-activity dependent transcription.^175^ CRTCs have also been suggested to be involved in ACTH-induced transcription of StAR (Steroidogenic Acute Regulatory) protein, where ACTH mediates the recruitment of CRTC2 and CRTC3 to the StAR promoter leading to increased levels of Star heteronuclear RNA,^176^ indicating that these co-activators modulate context-specific activation of diverse genes.

### CITED family

CITED (CBP/p300-interacting transactivators with E (glutamic acid)/D (aspartic acid)-rich carboxyl-terminal domain) family of transcriptional co-activators are 22–27 kDa proteins^177^ that interact directly with the CBP/p300 family of transcriptional co-activators through a conserved C-terminal domain known as CR2 (conserved region 2).^178^ All the known members of CITED family undergo nuclear translocation where they interact with sequence-specific DNA binding proteins and activate transcription in a CBP/p300 dependent manner.^179^ CITED2 has also been reported to be essential for embryonic development.^180^ The embryonic fibroblasts of CITED2^-^/^-^ mouse had defective proliferation, senescence-associated cellular morphology and increase in expression of cell growth inhibitors p16^INK4a^, p19^ARF^, and p15^INK4b^.^181^ CBP/p300 interacts with HIF1α through its CH1 domain to activate transcription of hypoxia responsive genes and promote tumor angiogenesis. CITED2/CITED4 interacts with CBP/p300 at the CH1 domain, preventing association with HIF1α and functions as an inhibitor of hypoxia signaling.^182^ Accumulated evidence, therefore, indicates involvement of the CITED family in various biological activities to regulate CBP/p300-dependent transcription.

### TRIM family

Tripartite motif-containing (TRIM) protein super family is associated with a wide range of biological processes.^183^ The TRIM motif (also known as RBCC motif), which identifies this superfamily, consists of a RING domain, one or two B-box domains, N-terminal-associated coiled-coil domain and C-terminal domain. In humans, ~70 TRIM genes have been identified which have been further subclassified on the basis of their C-terminal domain.^183^ The RING domain mediates conjugation with ubiquitin, with SUMO (small ubiquitin-like modifier), or with ISG15 (IFN-stimulated protein of 15 kDa).^184^ The zinc-binding motif containing RING domain, is the catalytic center which provides biological flexibility to the TRIM family of proteins.^185^ The RING domains of TRIM5α, TRIM8, TRIM11, TRIM21, TRIM22 and TRIM25 mediate ubiquitylation events owing to the E3 ubiquitin ligase activity.^186^ This E3 ubiquitin ligase activity has been established to be crucial for anti-HIV functions.^184^ Some members of the TRIM family contain a COS box which is located immediately downstream of the coiled-coil domain. The COS box mediates binding to microtubules.^187^ C-terminal domains, like the ADP ribosylation factor-like (ARF) domain, are associated with vesicular trafficking, whereas fibronectin type 3 (FN3) domains, might be involved in actin crosslinking. Owing to the presence of bromodomain, the TRIM family members (TRIM24, TRIM28, and TRIM33) can act as chromatin remodelers.^188^ An example of the kind is the regulation of self-renewal transcription network by TRIM28. TRIM28, together with other pluripotency markers like CNOT3, ZFX, and c‐MYC, co-occupies putative gene promoters to promote self-renewal.^189^ TRIM24-mediated regulation of glioma stem cell proliferation and self‐renewal has also been reported. In response to EGFR, TRIM24 recruits STAT3 and stabilizes STAT3-chromatin interaction to promote cancer stem cell proliferation and maintenance.^190^ Given their role as transcriptional co-activators, the TRIMs have the potential to emerge as therapeutic targets in different pathological conditions.

### MRTF family

The mechano-sensitive myocardin family of transcriptional co-activators comprising myocardin, MRTF-A/MKL1/MAL, and MRTF-B/MKL2 are associated with the MADS box transcription factor SRF (serum response factor) to activate transcription of genes responsible for myogenesis, cell proliferation, migration and creation of transcriptional–cytoskeletal regulatory circuit by encoding components of actin cytoskeleton.^191^ The MRTFs contains several conserved domains that are essential for actin-binding, chromatin organization, homo- and hetero-dimerization and transcriptional activation.^192^ Esnault et al.^193^ identified 960 serum-responsive SRF-linked genes and majority of these genes were regulated by MRTF-mediated RNA polymerase recruitment and promoter escape. In the context of pathology of the intervertebral disc (IVD), Fearing et al.^194^ reported that transcriptional co-activator MRTF-A regulates nucleus pulposus cell phenotype. MRTF-A and transcription co-activators YAP/TAZ promotes pathologic and fibroblastic phenotype of the adult human NP cells in association with F-actin stress fibers, indicating that the MRTF-family of co-activators are principal regulators of cytoskeletal dynamics and mechano-sensing, both under normal and diseased physiological conditions.

### DExD/H box family

The DExD/H (Asp-Glu-x-Asp/His) box family of proteins are known to play major roles in RNA synthesis and function.^195^ Owing to the homology with DNA helicases, the prototypic members of the family exhibits ATP-dependent RNA helicase activity. These proteins also act as RNA chaperones and promote local RNA unwinding to mediate the formation of optimal RNA structures.^196^ The DExD/H proteins contain N- and C-terminal extensions through which they interact with several components of the transcriptional machinery to regulate transcription. For instance, DDX5 (p68) has been demonstrated to act as transcriptional co-activator of Polo-like kinase-1 (PLK1) by stimulating the transcription from PLK1 responsive promoter.^197^ DHx9 interacted with CBP with its N-terminal domain, while the helicase domain and an overlapping region of the N-terminal domain was found to interact with Pol II, thereby enhancing the enforcement of the transcriptional complex at responsive promoters.^198^ DDX3 has been depicted to co-activate transcription from p21 promoter.^199^ Furthermore, DDX3 also facilitates the interaction of IRF3 with the transcriptional co-activators CBP/p300, hence guiding an antiviral signaling-induced transcription factor complex formation on target gene promoters.^200^ Owing to the accelerating importance of DExD/H-box family of proteins in transcriptional regulation, further descriptive studies to decipher their significance in normal physiology and disease pathology, may provide alternate therapeutic options.

### PGC-1 family

The members of peroxisome proliferator-activated receptor γ (PPARγ) coactivator 1 (PGC-1) family of transcriptional co-activators have been reported to exert several biological functions like energy metabolism, skeletal muscle fiber type switching, heart development, adaptation to thermogenesis, and endurance-type exercise.^201,202^ The founding member of this family is PGC-1α. This small family of co-activators also includes PGC-1β, the close homolog of PGC-1α and PGC-1-related coactivator (PRC).^203^ The N-terminus contains the major nuclear hormone receptor-interacting motif (LXXLL), which facilitates ligand-dependent interactions with different transcription factors like ER,^204^ PPARα,^205^ RXRα,^206^ glucocorticoid receptor and HNF4α.^207^ The C-terminal region contains the RNA processing motifs like the serine-arginine-rich (RS) domain and a RNA-binding motif (RMM). The presence of the transcription activation domain along with the RNA processing motifs is a unique feature of the PGC-1 family.^73^

PGC-1 family of proteins acts as secondary co-activators by serving as a docking platform for other co-regulatory molecules.^30^ In humans, PGC-1α is a master regulator of energy metabolism and mitochondrial homeostasis. PGC-1α co-activates the expression of nuclear respiratory factors 1 and 2 (NRF1 and NRF2) which further facilitates the transcription of genes associated with mitochondrial respiratory chain complexes.^208^ Human PGC-1α also interacts directly with RNA and with NXF1 (Nuclear RNA export factor 1) to promote nuclear export of co-activated transcripts, essential for age-related telomere maintenance.^209^ PGC-1β has been reported to upregulate expression of genes associated with oxidative phosphorylation and electron transport chain.^210^ Moreover, PGC1β KO mice demonstrated decreased activity during the dark cycle and less response to physiological stresses, like adrenergic stimulation in BAT (brown adipose tissue), cold exposure in BAT, and hepatic steatosis.^211^ Altogether it can be stated that the PGC-1 family of co-activators play a non-redundant role in the basal and stress-related mitochondrial activity regulation.

## Regulation of transcriptional co-activators

### Regulation of co-activator activity and expression

Activity and expression of co-activators can determine the fate of a cell by modulating an immensity of physiological processes.^212,213^ The state of normalcy in a cell is determined by the delicate maintenance of several essential factors, including the co-activators, failure of which will eventually lead to a diseased condition.^214,215^ The mechanisms for molecular regulation of these co-activators are described below.

#### Signal transduction

Transient signals induced by interactions of cell surface receptors and ligands are translated into prolonged alterations in the gene expression profile by various signaling pathways, entailing reversible assembly of numerous factors.^216,217^ These signal transductions control expression and activity of transcription factors, as well as co-regulators, thereby modulating cellular transcriptional program.^218^ Heretofore, countless studies have predicted the possibility of regulation of co-activators by signaling pathways. Willert et al.^219^ found CBP/p300 to be one of the target genes of WNT signaling pathway by microarray analysis. Moreover, CBP/p300 has been found to act as a co-activator of β-catenin, indicating towards a possible feedback loop mechanism.^220^ 27 of the 72 TRIM family genes are reported to be sensitive to interferon signaling.^221^ In skeletal muscles, PGC-1α activity is governed partly by p38 MAPK and CaMKII^222^ and in liver by LIPIN1.^223^ Another study reported PGC-1α is regulated by TLR2 signaling in mice with *Staphylococcal aureus* sepsis.^224^ In head and neck cancer, the WNT pathway effector protein, β-catenin, was found to play important role in MLL1 transcription regulation.^225^ In diabetic nephropathy, transcription co-activator SET7 is regulated by the TGF-β pathway.^226^ Multiple studies have reported the hippo signaling pathway to be the prime regulator of YAP/TAZ expression and activity.^227^

#### Epigenetic regulation

The genome of all the cells in an organism essentially consists of the same DNA. However, their functions vary depending on the quantitative difference in their gene expression profile.^228^ This form of regulation renders an additional adaptive switch that helps the organism to exquisitely regulate expression and function of different factors and sustain under unfavorable conditions.^229^ Activity of transcriptional co-activators has also been documented to be regulated by such epigenetic modulations.^230^ For example, YAP is monomethylated at lysine 494 by another co-activator SET7, which helps in cytoplasmic retention of YAP.^231^ Methylation at arginine residue of KIX domain of CBP by coactivator-associated arginine methyltransferase 1 (CARM1) inhibits the interaction of CBP with CREB, thereby, blocking their downstream activity.^232^ Rieger et al.^233^ proved that phosphorylation of p300 at serine 89 by protein kinase C (PKC) regulates its interaction with β-catenin.^234^ It was observed that in early anaphase, cyclin dependent kinase-1 (CDK1)/Cyclin B complex stabilizes SET7 by phosphorylation at the serine 29 residue. In addition, acetylation of MLL1 at two conserved residues, K1130 and K1133, by sirtuin1 (SIRT1) affects its methyltransferase activity.^235^ Liu et al.^236^ found that BRD4 methylation at R179, R181, and R183 residue by protein arginine methyltransferase2/4 (PRMT2/4) is essential to selectively control the transcriptional program by facilitating BRD4 recruitment to histones or chromatin. Regulation of a co-activator activity by another co-activator, where BRD4 was found to be methylated at lysine 99 residue by SETD6, which in turn negatively regulates target gene expression, was also reported.^237^ Activity of transcription co-activator TRIM5α is restricted by autoubiquitination, wherein, E2 Ub-conjugating enzyme Ube2W is employed to anchor the Lys63-linked polyUb chains.^238^ A research article by Mersaoui et al.^239^ provided evidence that arginine methyltransferase 5 (PRMT5) methylates DDX5 at its RGG/RG motif by direct interaction. This motif is necessary for DDX5 to interact with XRN2 and repress formation of cellular R-loops, which is essential for transcriptional termination. In accordance, Wu et al.^240^ proposed a unique regulation of SRC3 by a coordinated phosphorylation dependent ubiquitination mechanism.

#### Protein–protein interactions

The context-dependent activation and inactivation of transcription co-activator function is often determined by the proteins they interact with. BRD4, for example, interacts with different proteins under specific circumstances and therefore, regulate multiple cellular pathways.^241^ Mechanistically, Yu et al.^241^ revealed that in hepatocellular carcinoma, DDX5 forms transcriptional regulatory complex in association with BRD4 to positively regulate transcription of phosphatidylinositol-4,5-bisphosphate 3-kinase catalytic subunit alpha (PIK3CA). BRD4, via its extra-terminal domain, interacts with arginine demethylase Jumonji domain-containing 6 (JMJD6), lysine methyltransferase nuclear receptor-binding SET domain 3 (NSD3), and the nucleosome remodeling enzymes SWIF/SNF and CDH4, to perform context specific functions.^242^ The Human Papillomavirus Type 16 E6 oncoprotein physically binds to CBP/p300 and downregulates p53 transcriptional activity.^243^ PIMT (PRIP-interacting protein with methyltransferase domain), a RNA-binding protein, strongly binds to CBP/p300 through its cysteine-histidine rich C/H1 and C/H3 domains and regulate their activity.^244^ Sheppard et al.^245^ unveiled the importance of the interaction between CBP/p300 and SRC1 through its activation domain 1 (AD1) in assisting the recruitment of CBP/p300 to the estrogen receptor.

#### Non-coding RNAs

Non-coding RNAs (ncRNAs) are functional RNA molecules that do not have protein coding region, and are therefore not translated into protein.^246^ However, they actively take part in expression and activity regulation of diverse proteins, including transcription co-activators.^247^ Most extensively studied ncRNAs in co-activator modulation are microRNAs (miRNAs) and long non-coding RNA (lncRNAs).^248^ MicroRNAs generally regulate co-activator gene expression by direct interaction with the mRNA but their role in activity regulation is not explicitly understood.^249^ However, lncRNAs can regulate both co-activator activity and expression owing to their diverse mode of action.^250^ There are multitudinous studies unveiling the interplay between co-activators and ncRNAs. For instance, sequencing (ChIRP-seq) together with CRISPR/Cas9 mutagenesis of the target sites proved that p300 is recruited to the enhancer region by lncSMAD7 to trigger enhancer acetylation and transcriptional activation of its target gene.^251^ Lagos et al.^252^ showed that miR-132 suppresses p300 activity during antiviral innate immune response. NEAT1 lncRNA forms a complex with BRD4 and WDR5 and maintains them in a less-active state.^253^ In multiple myeloma, dual luciferase reporter assay showed that H19 inhibits miR-152-3p to enhance BRD4 expression.^254^ There is also evidence suggesting negative regulation of BRD4 by miR-141-3p.^255^ In ovarian cancer, SET7 has been shown to be modulated by miR-153, and lncRNA SNHG6 has been found to downregulate SETD7 by posttranscriptional destabilization.^256^

### Modulation of co-activator function by signaling pathways

Signal transduction pathways can be defined as coordinated interdependencies amongst structurally and functionally diverse class of biomolecules that conjointly dictate the response of a given cell to a particular cue received by endocrine, paracrine and cytokine signaling.^257^ The preponderance of transcriptional co-activator-related research articles and knowledge bases has recognized them as one of the pivotal molecules that are being actively regulated by signaling pathways. Gusterson et al.^258^ demonstrated that in cardiac cells, activation of CBP/p300 upon phenylephrine (PE) treatment is dependent on p42/p44 MAPK pathway. CBP/p300 has been reported to be degraded by murine double minute 2 (MDM2) in NIH-3T3 cells, which is regulated by the MAPK pathway.^259^ In addition, under certain circumstances, MAP4K downstream kinase nuclear dbf2-related 1/2 kinases (NDR1/2) directly phosphorylate and inhibit YAP.^260^ YAP has also been found to be ubiquitinated and degraded by PARK2, an important downstream factor of PLCE1-SNAIL axis.^261^ PYGO2, a WNT signaling downstream protein, facilitates the recruitment of MLL1/MLL2 complex to WNT target gene promoters.^262^ Transcriptional co-activator TRIM37 activation during Hepatitis B virus (HBV) infection-associated hepatic fibrosis, is mediated by reactive oxygen species (ROS)-induced nuclear factor κB (NF-κB) signaling.^263^ Meerson et al.^264^ have shown that leptin and insulin signaling indirectly modulates nuclear receptor co-activator 1 (NCOA1) through miR-4443. During prostate cancer progression, SRC-1 is phosphorylated by MAPK on Ser1185 and Thr1179 and thereby, increases its binding affinity to androgen receptors (AR).^265^ p38 MAPK and GSK3 have also been reported to phosphorylate SRC-3 on ser869 and ser505, which not only enhances its binding ability with AR but also determines the mode of action through ubiquitination.^240^ GSK3β has been observed to negatively regulate PGC-1α through inhibition of transcription factor EB (TFEB), which has an established role in PGC-1α gene expression.^266^ Puigserver et al.^267^ discovered many cytokines that stimulate activating phosphorylation of PGC-1 through p38 MAPK pathway, ultimately resulting in heightened respiration and energy expenditure in muscle cells. One of the most significantly upregulated miRNAs in response to elevated WNT signaling cascade, miR-150, is found to markedly suppress CREB signaling pathway by targeting its core transcription factors CREB1 and EP300.^268^ Jun N-terminal kinase (JNK) inhibits CRTC3 activity by mediating their phosphorylation and cytoplasmic retention.^269^ The AMPK signaling is another well-known phosphorylation-dependent inducer of CRTC activity.^270^ These context-dependent diverse modes of regulation of transcription co-activators provide a rational platform for effective disease diagnosis and therapeutics.

### Interplay between co-activators and co-repressors

Cellular homeostasis is maintained by a perplexing complexity of transcriptional networks that regulate gene expression programs within a cell.^271,272^ The cycling behavior of the transcriptional network that alternates between on/off stages balances the transcriptional output. The key regulators of these alternate cycling events are the transcriptional co-activator and co-repressor molecules that function as “accelerator and brake”, respectively, to control target gene expression, in association with specific transcription factors.^273^ The transcriptional active and inactive states are significantly reinforced through different mechanisms like acetylation/deacetylation and methylation/demethylation, which are mediated by the collaborative interplay between transcriptional co-activators and co-repressors in relation to cell-specific chromatin contexts.^274^ In a normal physiological system, the dynamic equilibrium between the expression of transcriptional co-activators and co-repressors controls transcriptional plasticity, to regulate waves of transcription cycling which delicately equipoise homeostasis.^275^ One such example is of the thyroid hormone (TH)-mediated gene transcription. The thyroid hormone receptors (TRs) can bind to thyroid hormone response elements (TREs) in both liganded and unliganded conformation. When bound to TH, the receptor undergoes a conformational change that promotes the recruitment of transcriptional co-activators with histone acetyl transferase (HAT) activity that generates a permissive chromatin environment to promote target gene expression. However, in the absence of TH, due to a different structural conformation in the unliganded state, the TRs recruit a co-repressor complex (Co-R) with histone deacetylase activity (HDAC) that induces a repressive chromatin environment to prevent transcription of target genes. Thus, the co-ordinated action of transcriptional co-activators and co-repressors tightly control the TH-mediated gene transcription in cells.^276^ Another recent study conducted by Zaghet et al.^277^ has revealed that the interaction between the co-activators and co-repressors play an important role in preserving germ cell identity and immortality in *C. elegans*. H3K36 and H3K27 methylation propagated by methyltransferases is essential for germ cell maintenance. JMJD-5/KDM8, Jumonji C domain-containing demethylase/hydroxylase, which has been documented to function as context-dependent transcriptional co-activator or co-repressor,^278^ does not constrain H3K36me2 regions or remove H3K36me2 deposition. However, JMJD-5 blocks H3K36me2 accumulation in the regions that are normally associated with this modification. Therefore, a precise balance of methylation regulated by the methyltransferases and histone demethylates is essential for maintaining equilibrium.^277^

**Fig. 2:**
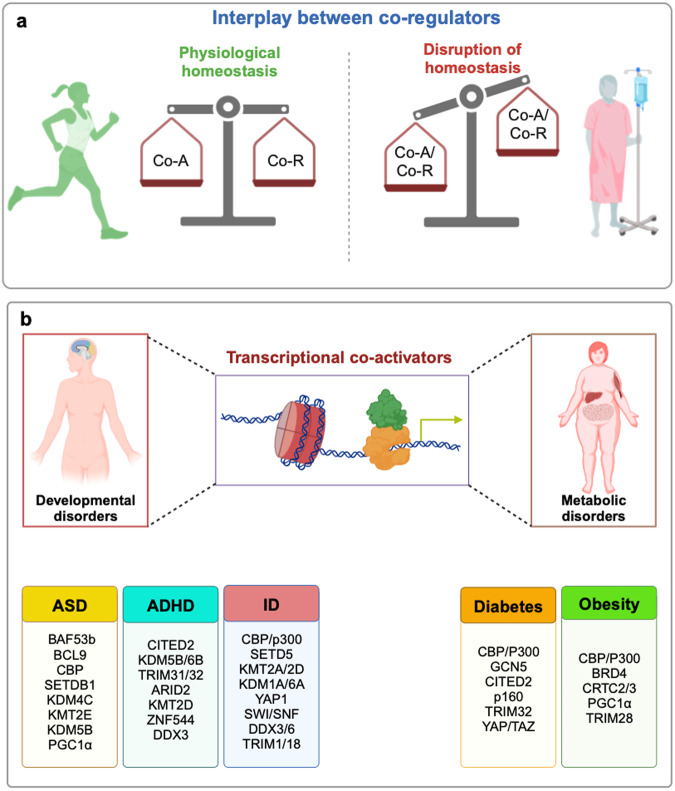
Transcriptional co-activators: Interplay with co-repressors and involvement in developmental and metabolic disorders. **a** In healthy individuals, cellular homeostasis is perpetuated by a dynamic equilibrium between the transcriptional co-activators (Co-A) and co-repressors (Co-R), that fine tunes the balance between cell proliferation and cell death signals. However, during disease conditions, like malignant transformation, the balance is skewed towards those co-regulators (both co-A and co-R) that mediate cell proliferation signals. Context-specific gain-of-function or loss-of-function mutations of transcriptional co-regulators mediate upregulation of oncogenic transcriptional signaling, thereby facilitating cancer promotion and progression. **b** Involvement of transcriptional co-activators in three common developmental disorders (ASD autism spectrum disorder, ADHD attention deficit/hyperactivity disorder, ID intellectual disability) and two of the most prevalent metabolic disorders, diabetes and obesity. This figure was created using BioRender (https://biorender.com/)

Contrary to the conventional regulatory mechanism, a myriad of evidences suggests that during malignant transformation, distorted transcriptional regulation is observed due to transcriptional rigidity.^279^ Cancer cell systems exhibit restricted plasticity due to which anti-mitotic inputs are disrupted, whereas the proliferative and anti-apoptotic signals are enhanced^280^ (Fig. 2a). For instance, the gain of function or loss of function mutations of transcriptional co-activators upregulate oncogenic transcriptional signaling, by facilitating permissive chromatin environment. One-third of cutaneous squamous cell carcinoma documents the loss of function mutations of CBP/p300 lysine acetyltransferases. Loss of function of these co-activators leads to enhanced Hras^S35^-mediated epidermal thickening, which initiates the formation of skin papillomas.^281^ However, gain of function of HAT/TAZ2 domain mutants have been observed in head and neck cancer patients. These CREBBP and EP300 mutations promoted a hyperacetylated state and enhanced DNA damage repair and radioresistance.^282^ Cancer progression also involves altered expression of transcriptional co-repressors. For instance, C-terminal binding proteins 1 and 2 (CtBP1 and CtBP2) are known to interact with polycomb group complexes, including components such as REST/CoREST, HDAC1 and HDAC2, to mediate transcriptional repression.^283^ However, CtBP1 is deregulated in malignancy. The elevated levels of CtBP expression across different cancer types have indicated that this co-repressor plays a key role in epigenetic regulation of cancer by repressing the transcription of a multitude of tumor suppressor genes.^284.285^ The loss-of-function of co-repressors has also been illustrated in oncogenic process. One such example is that of downregulation of the co-repressor breast cancer metastasis suppressor 1 (BRMS1). Loss of BRMS1 promotes carcinogenesis by facilitating the recruitment of RelA/p65 to NF-κB-dependent anti-apoptotic genes.^286^ Scaffold/Matrix-Associated Region-1 (SMAR1) deregulation in cancer is another example of co-repressor loss of function. Downregulation of SMAR1 promotes CCND1 transcriptional activation that promotes cancer cell proliferation.^287^ This deregulation of transcriptional co-regulators highlights the distortion of co-activator/co-repressor balance in disease pathology.

## Diseases associated with mutation of transcription co-activator families

Considering the compendium of previously stated facts on transcriptional co-activators, it is now quite evident that co-activators are indispensable for establishing homeostasis during gene expression. Therefore, exquisite regulation of these factors is imperative to maintain normal physiological conditions, derangement of which will cause manifestation of diseases. There are several reports that have delineated the mutations in these co-activator genes as the major force driving disease progression. Examples of diseases that are associated with the mutations of the co-activators have been summarized in Table 1. Involvement of co-activators in developmental disorders, metabolism-related diseases and cancer has been elaborated below.

**Table 1:** Diseases associated with mutations in the co-activator genes

Family	Gene	Diseases	Reference
CBP/P300	CBP	Rubinstein-Taybi syndrome, Huntington’s disease, Leukemia, Lung cancer, Colorectal cancer, Breast cancer, Head and neck cancer, Hepatocellular carcinoma, Gastric cancer	^282,580–587^
	P300	Rubinstein-Taybi syndrome, Leukemia, Lung cancer, Breast cancer, Head and neck cancer, Gastric cancer, Cervical cancer, Esophageal cancer	^580,588–591^
KMT	SETD2	Sotos syndrome or Luscan-Lumish syndrome (LLS)	^592^
	KMT2A	Wiedemann Steiner Syndrome (WSS), Leukemia	^471,593^
	KMT2D	Kabuki syndrome, Head and neck cancer, Colorectal cancer, Lung cancer, Diabetes	^594–598^
	KMT2B	Cerebral ataxia, Dystonia, Glioblastoma, Breast cancer	^599–602^
	KMT2C	Kleefstra syndrome, Colorectal cancer, Breast cancer, Leukemia, Lung cancer	^603–607^
SRC	SRC1	Prostate cancer	^608^
	SRC2	Prostate cancer, Lung cancer, Melanoma	^609,610^
DDX	DDX41	Myelodysplastic syndromes (MDS), Acute myeloid leukemia (AML), Cytopenia	^611–613^
	DDX3	Head and neck cancer, Medulloblastoma, Leukemia	^614–616^
TRIM	TRIM20	Multiple sclerosis, Alzheimer’s disease	^617,618^
	TRIM37	Mulibrey nanism	^619^
	TRIM19	Schizophrenia	^620^
	TRIM 32	Bardet-Biedl syndrome	^621^
PGC	PGC1α/β	Type II diabetes	^622^
BET	BRD4	Cornelia de Lange syndrome, Nephrocalcinosis	^623,624^
CITED	CITED2	Congenital heart disease	^625^
CRTC		Hereditary Pancreatitis	^626^

### Co-activator involvement in developmental disorders

Developmental disorders are known to be heterogeneous conditions that have been reported to affect a significant population of children worldwide.^288^ The most frequently diagnosed developmental conditions throughout the world are, autism spectrum disorders (ASD), attention-deficit/hyperactivity disorder (ADHD) and intellectual disability (ID).^289,290^ Wealth of evidences have suggested that chromatin remodeling and transcriptional regulation plays a crucial part in the development of these diseases.^291^ Here we have briefed the co-activator mediated transcriptional deregulations that lead to developmental disorders (Fig. 2b).

#### Autism spectrum disorder (ASD)

A component in mammalian SWI/SNF complex, BAF53b is essential for neuronal development, function and cell identity.^292^ Loss of function of BAF53b has been associated with increased risk of developing ASD.^293^ BCL9 and CBP deletion have also been reported in ASD.^294^ De novo mutation leading to an amino acid substitution of the transcriptional co-activator MKL2 or MRTFB has been associated with ASD. However, the mechanism of this mutation mediated AD development is yet to be elucidated.^295^ SETDB1 has been shown to influence embryological development by promoting the maintenance of pluripotency and suppressing the differentiation of embryonic SCs,^296^ and therefore, is required for nervous system development and function while dysregulation of SETDB1 is implicated in the pathogenesis of CNS disorders including ASD.^297^ Altered expression or deletion of KDM4C has been linked to altered methylation patterns leading to autism.^298^ Another histone methyltransferase KMT2E (MLL5) haploinsufficiency has been linked to manifestation of autism like behavior in mice.^299^ 3% of individuals with ASD were found to exhibit multiple de novo frameshift insertion and deletion mutations in this gene. Moreover, a cohort of 2500 patients has been reported to contain de novo missense and nonsense mutation of histodemethylase KDM5B.^300^ Missense variant dead box helicase 5 (DDX5) have been shown to affect protein-protein interactions and, increase the risk of ASD.^301^ A study by Crider et al.^302^ provided evidence that a significant decrease in the expression of ER co-activators, SRC1 (34%), CBP (77%), PCAF (52%) was observed in the middle frontal gyrus of ASD patients. Benito et al.^303^ found that pharmacological inhibition of BET/BRD leads to autism-like behavior in mice. A significant proportion of ASD cases have been observed to possess mitochondrial metabolic dysfunction.^304^ Hypermethylation of PGC-1α promoter-induced mitochondrial dysfunction has also been found to cause ASD.^305^ Hence, it can be stated that the co-activator molecules have a critical role in autism spectrum disorder (ASD) and therefore, can be a good therapeutic target for amelioration of ASD and related diseases.

#### Attention deficit/hyperactivity disorder (ADHD)

Transcription co-activator CITED2 has been found to contribute in maintaining proper somatosensory neocortical length, neuronal connectivity, and neocortical development.^306^ Conditional knockout of CITED2 in the forebrain of mice led to aberrant neocortical development, which can be associated with ADHD.^307^ Gao et al.^308^ proved that haploinsufficiency of KDM6B can be linked to ADHD related behaviors in mice. Olfson et al.^309^ conducted whole exome sequencing of 152 parent–child trios and identified KDM5B to be one of the high-risk genes in ADHD. Rare copy number variation in TRIM32 gene and single nucleotide polymorphism of TRIM31 gene are the drivers of ADHD development.^310^ Mutation in a SWI/SNF chromatin remodeling complex protein ARID2, has been found in the patients with ADHD.^311^ Analyzing whole-genome sequencing data from 272 patient samples, Zhou et al.^312^ showed that one of the top candidate genes that are linked with ADHD is KMT2D. An epigenome-wide association study revealed the association of co-activator ZNF544 with ADHD during early childhood.^313^ Geneviève et al.^314^ found that 44% of the individuals with DEAD-box RNA helicase 3 (DDX3)-related disorders suffer from attention deficit/hyperactivity disorder (ADHD) symptoms. Though, evidence indicating the connection between co-activator deregulation and developmental disorders is abundant, very few, if any, systemic study deciphers the detailed molecular mechanism.

#### Intellectual disability (ID)

Acetylation status of proteins is exquisitely regulated in neuronal plasticity and cognition behavior regulation. One of the main regulators of this status, CBP/p300, has been found to have a link in ID progression.^315^ Mutation at 3p25.3 on SETD5 gene, which is expressed throughout the brain, is suggested to facilitate ID.^316^ KMT2D and KDM6A gene mutations lead to defective methylation pattern and as a consequence, drive Kabuki Syndrome-related ID.^317^ Lebrun et al.^318^ studied KMT2A gene in a cohort of 200 patients and found deletion and missense mutation in Wiedemann-Steiner syndrome related IDs. Mutations in lysine demethylase 1A (KDM1A) affect their active site residues and catalytic activity, which in turn limits their binding affinity to TFs. These mutations are reported to promote intellectual ability impairment.^319^ YAP1 loss-of-function mutations were observed in patients with Colomoba, an eye abnormality that is often associated with intellectual disability.^320^ A rare neurodevelopmental disorder caused by variation in the genes, encoding members of SWI/SNF family of transcriptional co-activators, is SWI/SNF-related intellectual disability disorders (SSRIDDs). The most common cause of SSRIDD is mutation in ARID1B, which is a core component of SWI/SNF complexes.^321^ Barish et al.^322^ reported that SSRIDDS is also associated with mutations in BICRA (BRD4 interacting chromatin remodeling complex-associated protein) gene. Similar to ADHD, mutations in DDX3X have been associated with intellectual disability. Blok et al.^323^ reported that in females, mutations in DEAD box helicase protein DDX3X accounts for 1–3% of unexplained intellectual disabilities. De novo mutations and segregating missense mutations were also observed in males. Through their study Blok et al.^323^ established that DDX3X mutation possess an X-linked recessive inheritance pattern. Balak et al.^324^ further reported that de novo missense mutation of DDX6 is also associated with intellectual disbility. X-linked intellectual disability (XLID) contains TRIM1 missense mutations (p.R347Q and p.N343S) in affected as well as obligate carriers. Moreover TRIM1 mutation (p.Asn343Ser) was found in 480 patients with idiopathic intellectual disability,^325^ whereas mutations in TRIM18 led to X-linked form of Opitz Syndrome.^326^

### Co-activator involvement in metabolic disorders

Metabolic disorders can be described as a constellation of intertwingled pathophysiological abnormalities arise from metabolic origin.^327^ The most commonly occurring metabolic disorders are diabetes and obesity.^328^ The need for identification and characterization followed by therapeutic implementation is also rising. Metabolic disorders are genetically diverse disease and a myriad of gene regulation complexes have been linked with it.^329^ Transcription co-activators are one of the multiple factors that closely govern the process of transcription and metabolic disorder progression.^330^ Here we have summarized the co-activators that are reported frequently in the context of diabetes and obesity (Fig. 2b).

#### Diabetes

In the last few decades, diabetes has been emerged as one of the most diagnosed metabolic disorders with almost 463 million cases worldwide. Progressive loss of β-cell identity and insulin resistance is generally associated with type 2 diabetes.^331^ It has been observed that downregulation of CBP/p300-mediated H3K27 deacetylation promoted β cell failure in type 2 diabetes in islets of prediabetic *db/db* mice.^332^ Moreover, in hyperglycemia, loss of p300 histone acetyl transferase activity promotes β cell apoptosis.^333^ However, unbalanced levels of histone acetylation have been found to be involved with diabetic retinopathy, one of the major causes of diabetes-associated morbidity. Significant increase in acetylation of retinal histone H3 at lysine 9 (H3K9) and lysine 23 (H3K23) was observed in experimental diabetic animals. It was also observed that in the retina, HAT p300-mediated acetylation is associated with proinflammatory molecule induction, suggesting that transcriptional co-activator-mediated acetylation is a major contributor of diabetic retinopathy^334^; hence, a tissue-specific role of CBP/p300 is predominant in diabetes manifestation. Sakai et al.^335^ further established that disruption of the GCN5 and CITED2 ameliorates diabetes and also dampens gluconeogenesis. The p160 co-activators (p/CIP and SRC-1) have also been found to negatively regulate insulin sensitivity and the levels of insulin receptor substrate (IRS) proteins. Moreover, downregulation of p/CIP and SRC-1 was found to enhance insulin sensitivity and increase glucose uptake in both regular and high fat diet-fed p/CIP and SRC-1 double knockout (DKO) mice,^336^ indicating that targeting these diverse co-activators, is a promising pharmacological target for treatment of both type 2 diabetes and obesity. Role of TRIM family of transcriptional co-activators has also been implicated in diabetes mellitus. Wan et al.^337^ reported that when compared to healthy control, elevated expression of TRIM32 was observed in the type 2 diabetes mellitus patients. In vitro experiments further revealed that under high glucose conditions, marked increase in the expression of TRIM32 along with a concomitant downregulation in the AKT and mTOR phosphorylation levels was observed, which further exacerbated pancreatic cell autophagy and hampered insulin secretion, thereby promoting development of type 2 diabetes. The Hippo pathway transcriptional co-activators YAP/TAZ has also been documented to mediate insulin resistance by promoting phosphorylation of IRS1. Combination treatment with YAP/TAZ inhibitor (verteporfin) and metformin led to complete inhibition of the insulin and IGF1 signaling.^338^ Collectively, more elaborate studies are essential to discern the role of transcriptional co-activators in diabetes, which will direct the development of new therapeutic strategies in future.

#### Obesity

Obesity is typically defined as a multifactorial chronic disease with several causes resulting in excessive body fat accumulation, which sometimes is associated with poor health conditions.^339^ Zhou et al.^340^ found that selective inhibition of the HAT domain of CBP/p300 histone acetyltransferases, by A-485, markedly decreased the fat mass in obese mice. Contrarily, another study reported that the loss of CBP in the hypothalamus resulted in obesity.^341^ Hu et al.^342^ predicted a mechanism of BRD4-induced obesity through peroxisome proliferator-activated receptor ɣ (PPARɣ)-dependent growth differentiation factor 3 (GDF3) regulation. Obese conditions in mice has been observed to activate CRTC2/3 by decreasing the expression of salt-inducible kinases (SIK), a Ser/Thr kinase that phosphorylates and inhibits CRTCs.^343^ Tumor necrosis factor α (TNF-α) mediated PGC-1α downregulation has been reported in obesity in rodents. Similar reduction is also reported in obese human patient samples.^344^ Deletion of TRIM 28 and deficiency of SRC1 has also been associated with obese condition.^345^

### Co-activator involvement in different cancers

#### A rampant occurrence

With the rising burden of cancer, it has become imperative to develop new therapeutic approaches to curb disease progression. According to Globocan (2020), based on estimated worldwide age-standardized mortality rates, including all gender and all ages, lung cancer (18%), breast cancer (13.6%), colorectal cancer (9%), liver cancer (8.7%), stomach cancer (7.7%), prostate cancer (7.7%), cervical cancer (7.3%), esophageal cancer (5.6%), pancreatic cancer (4.5%) and ovarian cancer (4.2%) accounts for a substantial amount of cancer-related deaths.^346^ Moreover, based on age-standardized mortality rates, head and neck cancer (including lip, oral cavity, larynx, oropharynx, hypopharynx, salivary glands and nasopharynx) and leukemia, contributes to 8.59% and 4%, respectively, of the total cancer-related deaths.^346^ Hence, with the rising burden of cancer, it has become imperative to develop new therapeutic approaches to curb disease progression. Based on the Globocan statistics, this review attempts to abridge the involvement of several transcriptional co-activators that are responsible for the deregulated activity of several transcription factors across these twelve cancer types, which contribute substantially to cancer-related mortality worldwide. Such an approach will consequentially unearth novel therapeutic targets to curtail tumor progression, thereby reducing the burden of cancer.

#### Co-activator involvement in the deadliest forms of cancer

##### Breast Cancer

**Fig. 3:**
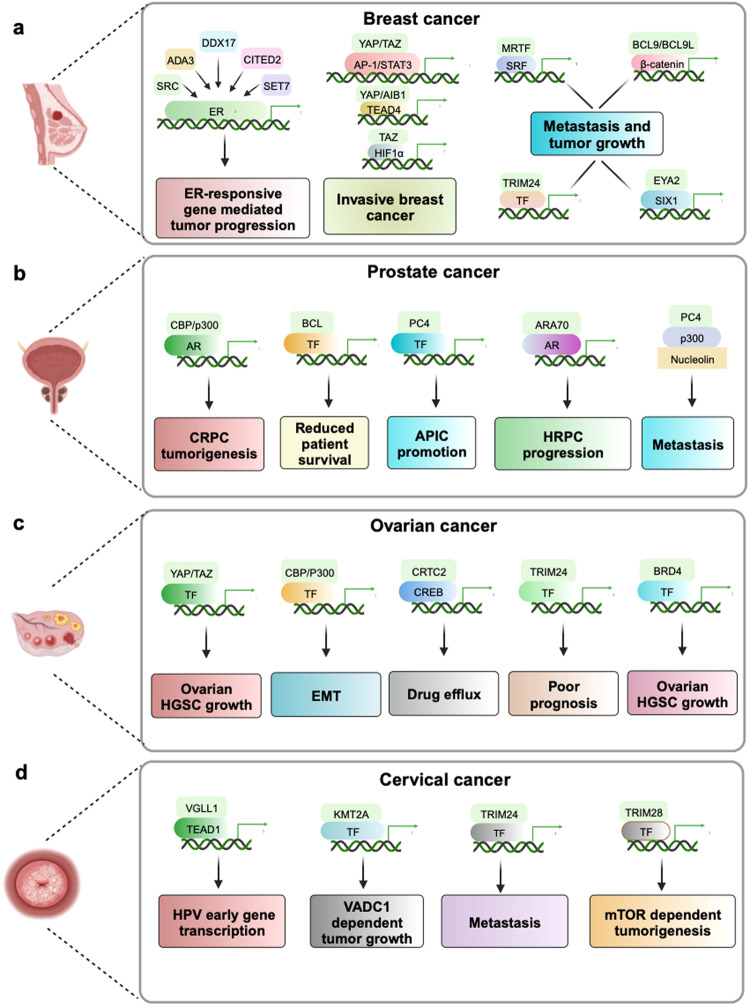
Transcriptional co-activators effectuate transcription of oncogenes in cancers of endocrine organs. (**a**) Several transcriptional co-activators, namely SRC, CITED2, SET7, DDX17, and ADA3 have been identified to work in association with the hormone-regulated transcription factor, estrogen receptor (ER), to promote breast cancer. Apart from ER co-regulators, several other transcriptional co-activators like MRTF, BCL9/BCL9L, EYA2, TRIM24 and YAP/TAZ promote breast cancer tumorigenesis. (**b**) In prostate cancer, the involvement of transcriptional co-activators CBP/p300,BCL9, PC4and ARA70 have been documented. (**c**) Enhanced expression of YAP/TAZ, CBP/p300, CRTC2, TRIM24, and BRD4 have been observed to be associated with ovarian cancer metastasis, therapy resistance and poor patient prognosis (**d**) The coordinated interaction between VGLL1 and TEAD1 promotes cervical cancer growth by mediating transcription of HPV early genes. Other transcriptional co-activators associated with cervical cancer are KMT2A, TRIM24 and TRIM28. CRPC, castrate-resistant prostate cancer; APIC, androgen-independent prostate cancer; HRPC, hormone-refractory prostate cancer; HGSC, ovarian high-grade serous carcinoma. This figure was created using BioRender (https://biorender.com/)

Breast cancer has been reported to be the most commonly diagnosed cancer with nearly 2.3 million new cases in the year 2020 (Globocan, 2020).^346^ In addition, breast cancer is the leading cause of cancer related deaths in women worldwide.^347^ Diverse histopathological subtypes make it more difficult to predict the progression of the disease. Hence, despite the progress in its detection and treatment, it seems necessary to unravel the roots of breast cancer so that new therapeutic approaches can be designed for its proper abrogation^348^ (Fig. 3a).

#### MRTF

Myocardin-related transcription factors (MRTFs) are a family of functionally related transcription co-activators that include myocardin, MRTF-A/MKL1/MAL and MRTF-B/MKL2, etc. This family of proteins associate with the MADS box transcription factors like serum response factor (SRF) and induce transcription of genes responsible for Rho-dependent cytoskeletal processes like cell motility, adhesion, and spread of breast cancer cells.^349^ Microtubule-associated serine/threonine-protein kinase-like (MASTL) acts as an activator of MRTF-A/SRF (myocardin-related transcription factor A/serum response factor) signaling. Taskinen et al.^350^ observed that mechanistically, MASTL associated with MRTF-A and increased its nuclear retention and transcriptional activity. This MASTL/MRTF-A signaling promotes breast cancer cell motility and invasion. Another study has reported that, P-cadherin upregulates MRTF/SRF signaling in early stages of breast carcinogenesis to promote self-renewal, proliferation and invasion.^351^

#### BCL9 and BCL9L

The aberrant activation of WNT/β-CATENIN signaling pathway leads to early events in carcinogenesis.^352^ Increasing evidences indicate that BCL9 and BCL9L transcriptional co-activators are over expressed in a significant population of breast cancer patients,^353^ and modulate the expression of β-CATENIN to promote tumor growth, cell migration, and metastasis in TNBC models.^354^ Targeting BCL9/BCL9L has been reported to have efficient anti-tumor effect through the inhibition of WNT and TGF-β signaling pathways, suggesting a viable therapeutic approach for TNBC treatment.^355^

#### SRC

The transcriptional activity of the estrogen receptor (ER) is regulated by its ligands as well as the co-regulators.^356^ Thus, changes in the expression of ER co-activators may be of utmost importance for the response to endocrine therapy.^357^ SRC (Steroid Receptor Coactivator) family of co-activator proteins including SRC-1, SRC-2, and SRC-3 are the most well-known ER co-regulators. SRC-1 and SRC-3 are of particular importance since high levels of these two transcriptional co-activators have been found in a number of breast cancer studies^358^ and their increased levels are associated with nodal positivity and endocrine resistance.^359^

#### CITED2

Another ER transcriptional co-activator in breast cancer is CBP/p300-interacting transactivator with Glu/Asp-rich carboxy-terminal domain 2 (CITED2) which is overexpressed in breast cancer tissues and is associated with worse clinical outcome.^360^ Increased expression of this protein may result in estrogen-independent ER activation, thereby reducing estrogen dependence and response to hormone therapies.^361^ Jayaraman et al.^362^ has also documented that CITED2 can modulate macrophage recruitment to influence breast cancer growth.

#### SET7

Yet another ERα transcriptional co-activator that has been recognized for several years is SET7, a protein lysine methyltransferase (PKMT) encoded by the SETD7 gene which is a key regulatory enzyme that mediates methylation of lysine residues of histone and non-histone proteins.^363^ SET7 has been documented to be over expressed in clinical breast cancer samples and the over expression of SET7 has been associated with tumor size, weight and expression of VEGF.^364^ SET7 stabilizes ER by methylating lysine 302 (K302) residue which is essential for recruitment of the transcription factor to its target genes and their transactivation.^365^ Gene Ontology (GO) analysis suggests that the ERα and SET7 co-activated target genes are primarily involved in the regulation of cell migration, but the precise molecular mechanism is undetermined till date.^366^

#### DDX17

The DEAD-box RNA helicase p72 (DDX17) has been shown to act as transcriptional co-activator for ERα.^367^ Studies have reported that knockdown of DDX17 results in a significant inhibition of estrogen-dependent transcription of endogenous ERα-responsive genes and estrogen-dependent growth of breast cancer cells.^368^ Moreover, in ER-positive breast cancer, DDX17 also acts as transcriptional co-activator of SOX2 and upregulates SOX2-mediated stem cell like features.^369^

#### ADA3

One of the kingpins that is associated with chromatin modification for transcriptional activation is the ADA3 (Alteration/Deficiency in Activation 3) protein which is an adaptor component of several lysine acetyltransferase complexes.^370^ Mirza et al.^371^ have reported that ADA3 is over expressed in breast cancer patients and as a transcriptional co-activator, human ADA3 (hADA3) interacts with ERα, thereby transactivating its downstream targets leading to breast cancer progression.^372^

#### EYA2

Amongst the different evolutionarily conserved transcription factors that impact carcinogenesis, sineoculis homeobox homolog (SIX) family proteins have been shown to play important roles in cell proliferation, migration, and apoptosis.^373^ SIX1, the most extensively studied of the SIX family members, is known to promote tumor invasion, metastasis, and paclitaxel resistance in breast cancer cells.^374^ In breast cancer patients, overexpression of co-activator EYA2 has been associated with poor prognosis.^375^ It has been reported that EYA2 (eyes absent 2) transcriptional co-activator, mandatorily forms a bipartite transcription initiation complex with SIX1 transcription factor and enhances proliferation, metastasis and DNA damage repair of breast cancer cells, ultimately promoting breast cancer progression.^376^

#### TRIM24

Tripartite motif 24 protein (TRIM24) also known as transcriptional intermediary factor 1α (TIF1α) is a transcriptional co-activator that has a N-terminal TRIM domain with three zinc-binding domains – a RING, a B-box type 1 and a B-box type 2 and also a coiled region with potential self-assembly properties,^377^ a C-terminal region containing a PHD finger, a bromodomain, and a nuclear receptor interaction box.^377^ TRIM24 transcriptional co-activator has also been shown to turnaround transcriptional networks to induce breast cancer progression and is associated with poor survival in breast cancer patients.^378^ It has been observed that TRIM24 directly activates *MET* gene expression, which in turn upregulates c-MET-PI3K-mTOR pathway in metaplastic breast cancer (MpBC).^379^ It has also been observed that TRIM24 interacts with SMAD3 and dissociates its interaction with tumor suppressor TRIM33. The TRIM24-SMAD3 complex is further recruited to chromatin, which enhances SMAD3 activation and immune response-related cytokine expression, thereby promoting enhanced breast cancer stemness, MDSC (myeloid-derived suppressor cell) recruitment, and metastasis.^380^

#### YAP/TAZ

The conventional wisdom suggests that YAP (Yes-associated protein) and TAZ (transcriptional coactivator with PDZ-binding motif) are transcriptional co-activators that majorly interact with TEAD family of transcription factors to promote tumorigenesis.^381^ However, recent evidences suggest, YAP and TAZ can also function as co-activators for AP-1 and STAT3 transcription factors leading to poor survival of triple negative breast cancer patients, but have meager effect on survival of patients suffering from other forms of breast cancers.^382^ Furthermore, TAZ can also act as a co-activator of hypoxia-inducible factor-1 (HIF-1α), which results in enhanced transcriptional activity of HIF-1α.^383^ Moreover, YAP in association with another transcriptional co-activator AIB1 (amplification of the p160 nuclear hormone receptor co-activator amplified in breast cancer-1; also known as NCOA3, SRC3, or TRAM3) has been shown to physically interact with TEAD4. This AIB1-YAP-TEAD4 interaction is essential for cell invasiveness in mammospheres.^384^

##### Prostate Cancer

According to Globocan (2020), a total of 1,414,259 new cases of prostate cancer and 375,304 prostate cancer-related deaths were reported globally. The increasing incidences of prostate cancer associated with the alarming mortality rates emphasizes the need to develop alternate therapeutic strategies to curb prostate cancer.^385^ The major co-activators involved are described (Fig. 3b).

#### CBP/p300

In primary and metastatic castration-resistant prostate cancer tissues, CBP/p300 are over expressed at mRNA levels.^386^ CBP/p300 has firmly been established to act as transcriptional co-activators of androgen receptor (AR).^387^ p300 has been documented to interact directly with AR N-terminal domain and AR-Ligand binding domain.^388^ Upon interaction, CBP/p300 acetylates AR and promotes AR stability.^389^ Ji et al.^390^ reported that CUB-domain-containing protein 1 (CDCP1) is highly expressed in late-stage and castrate-resistant prostate cancer (CRPC). In CRPC tumorigenesis, the co-activators BRD4 and CBP/p300 co-regulates the transcriptional activity of CDCP1.

#### CITED2

The transcriptional co-activator CITED2 has enhanced expression in metastatic prostate cancer and its expression is also correlated with poor survival. Shin et al.^391^ have reported that in prostate cancer, CITED2 acts as a molecular chaperone and guides p300 and PRMT5 to nucleolin, consequently inducing nucleolin activation. This CITED2-nucleolin axis is associated with prostate cancer metastasis.^391^

#### BCL9

BCL9 is highly expressed in clinical prostate specimens in comparison to the benign prostate tissues.^392^ In prostate cancer group, the positive rate of BCL9 was 53.1% (52/98), whereas in benign group the positivity rate was 25.0% (5/20; P = 0.022). Moreover, it was observed that the higher expression of BCL9 was correlated with shorter biochemical recurrence-free survival (P = 0.037) as indicated by Kaplan-Meier survival analysis.^393^ Detailed mechanism of the BCL9-mediated prostate cancer progression is yet to be elucidated.

#### PC4

The transcriptional co-activator PC4 is highly upregulated in prostate cancer and is associated with metastasis, progression and prognosis. PC4 has also been found to be significantly upregulated in androgen-independent prostate cancer (AIPC) when compared with Androgen-dependent prostate cancer (ADPC). It has been observed that PC4 suppress c-MYC/p21 pathway to inhibit cell growth and cell cycle arrest at G1/S phase. Moreover, PC4 also promotes the expression of HIF-1α and activates β-catenin signaling to exert its oncogenic activities.^394^ Further it has been recorded by Chakravarthi et al.^395^ that PC4 binds to the promoter region of several oncogenes like Polo-like kinase 1(PLK1), C-MYC, serine-threonine kinase BUB1B to regulate their expression.

#### ARA70

The first AR co-regulator that was identified is androgen receptor (AR)-associated coregulator 70 (ARA70). It has been observed that ARA70 interacts with ARA70-N2 domain via the consensus FXXLF motif to promote AR activity. Moreover, ARA70 is highly expressed in prostate cancer specimens (91.74%) than in benign tissues (64.64%, *p* < 0.0001). In addition, ARA70 is also upregulated in high-grade prostate cancer tissues and in hormone-refractory LNCaP xenografts.^396,397^ However, elaborate studies involving ARA70-mediated signaling has not been documented till date.

##### Ovarian Cancer

Ovarian cancer is one of the most common cancer in women and it accounts for 4.2% of total cancer related death in females. Though various reproductive and hormonal factors including parity, oral contraceptive use, and lactation may lead to lower risk, however several other causes like menopause at older age and hormone replacement therapy confer escalated risks of ovarian cancer.^398^ Therefore, identification of alternative therapeutic targets is necessary (Fig. 3c).

#### YAP/TAZ

The transcriptional co-activators YAP and TAZ have been reported to promote ovarian cancer tumorigenesis.^399,400^ Moreover, mRNA and protein levels of TAZ has been reported to be upregulated in ovarian cancer and a meta-analysis of microarray datasets of ovarian cancer has identified that increased expression of TAZ mRNA is correlated with poor prognosis in patients with ovarian cancer.^401^ Furthermore, YAP is highly expressed in inflammatory and cancerous fallopian tube tissues and YAP interacts with FGF-FGFR pathway to regulate fallopian tube umbilical epithelial cell activity. Recent studies have indicated that ovarian high-grade serous carcinoma (HGSC) might originate from fallopian tube umbilical epithelial cells primarily the secretory epithelial cells of fallopian tubes. Association of YAP with cancerous fallopian tube tissues further indicates involvement of YAP in HGSC.^402^

#### CBP/p300

Using the ovarian cancer cell line SKOV3, it has been reported that Staphylococcal nuclease domain-containing protein 1 (SND1) regulates the gene transcriptional activation of SLUG (an epithelial-to-mesenchymal transition marker) by increasing chromatin accessibility through the recruitment of the acetyltransferases GCN5 and CBP/p300 to the SLUG promoter proximal region.^403^ Moreover, physical association of BRCA1 was observed with the transcriptional co-activators/acetyltransferases p300 and CBP. Endogenous as well as overexpressed BRCA1 and p300 were found to associate in a phosphorylation-independent manner. BRCA1 interacts with the cAMP response element-binding protein (CREB) domain of p300/CBP via both its amino and carboxyl termini to mediate BRCA1-dependent transactivation.^404^

#### CRTC2

CRTC2 is over-expressed in chemo-resistant tissues of ovarian cancer. It has been observed that ovarian cancer patients with high expression of CRTC2 has poor prognosis. In addition, CRTC2 regulates the autophagic flux partially through PI3K-AKT pathway. Thus, CRTC2 might be a potential predictor as well as target for ovarian cancer.^405^ Furthermore, CRTC2 in association with CREB is also involved in the transcriptional activation of BCRP (Breast Cancer Resistant Protein)/ ABCG2, which further promotes ovarian cancer.^406^

#### BRD4

It has been observed that the transcriptional co-activator BRD4 is the fourth most amplified gene in HGSC, the most aggressive type of ovarian cancer. Overexpression of BRD4 has also been associated with poor patient prognosis. Moreover, increased expression of BRD4 is associated with upregulated expression of several oncogenes, such as MYC, NOTCH3, and NRG1. These oncogenes enhance tumor cell proliferation, genomic instability, metastasis, and resistance to chemotherapy.^407,408^

#### TRIM24

Zhang et al.^409^ have reported that TRIM24 is over-expressed in ovarian carcinoma in comparison to normal ovarian tissues. Upregulated expression of TRIM24 was observed to be closely correlated with serum CA-125 (P = 0.0294), metastasis (P = 0.0022), FIGO (International Federation of Gynecology and Obstetrics) stage (P = 0.0068) and Ki-67 level (P = 0.0395). Moreover, high expression of TRIM24 predicted worse prognosis in ovarian cancer patients. Furthermore, TRIM24 has been documented to promote AKT-phosphorylation, which in turn regulates metastasis. Another study by Zhou et al.^410^ has revealed that elevated expression of TRIM24 was linked to higher rates of lymphatic and distant metastasis. Moreover, TRIM24 negatively regulates the activity of FOXM1 to promote ovarian cancer progression.

##### Cervical Cancer

Cervical cancer, a malignant tumor of the lowermost part of the cervix, belongs to one of subsets of cancer with very high incidence and mortality rates. In the year 2020, the estimated number of new cases of cervical cancer was 604,000 with 342,000 deaths worldwide. It contributes to 7.3% cancer-related deaths in women worldwide.^346^ Identification of frequently deregulated factors is therefore necessary to design new therapeutic approaches to manage cervical cancer (Fig. 3d).

#### VGLL1

The key etiological agents responsible for the development of cervical cancer are human papillomaviruses (HPVs).^411^ It has been established that TEAD1 transcription factor activates the early promoter of human papillomaviruses.^412^ In addition, a study reported that TEAD1 mediated HPV early gene expression is regulated at the transcriptional level by VGLL1 (Vestigial-Like Family Member 1), which is a TEAD-interacting transcriptional co-activator. VGLL1/TEAD1 complex has been shown to interact with HPV16 long control region (LCR) and downregulation of VGLL1 and/or TEAD1 significantly decreases viral early gene expression, suggesting that VGLL1/TEAD1 is essential for efficient transcription of HPV early genes.^413^ Moreover, contrary to TEAD1, VGLL1 exhibits tissue-specific expression and is associated with development and differentiation of epithelial lineage tissues in concordance with HPV gene expression, thereby indicating that VGLL1 might facilitate epithelial specificity of HPV gene expression.^413^

#### KMT2A

KMT2A (histone-lysine N-methyltransferase 2A, former *MLL*) is a transcriptional coactivator with histone H3 lysine 4 (H3K4) methyltransferase activity.^414^ KMT2A is popularly known to be associated with acute leukemias, especially in infants, where it mostly interacts with six partner genes (*AFF1, MLLT3, MLLT10, MLLT1, ELL, AFDN*).^415^ However, a recent study has highlighted that KMT2A is also prevalent in cervical cancer, where it promotes cancer cell growth by regulating VADC1 (Voltage-dependent anion-selective channel 1). Downregulation of KMT2A was further shown to suppress cervical cancer cell proliferation and migration, accompanied with an activation of PARP/Caspase pathway and inhibition of VADC1, whereas overexpression of VDAC1 leads to a reversal of the KMT2A knockdown mediated changes, indicating that KMT2A/VDAC1 signaling axis might be a new therapeutic target for cervical cancer prevention.^416^

#### TRIM24

Cervical cancer has also been reported to have higher expression of TRIM24 transcriptional co-activator. It has been demonstrated that TRIM24 regulates the NF-κB and AKT signaling pathways, thereby contributing to cancer progression and metastasis.^417^

#### TRIM28

In comparison to their normal counterparts, cervical cancer cell lines and tissues also show an upregulated expression of TRIM28 transcriptional co-activator.^418^ TRIM28 has been found to significantly increase the phosphorylation of mTOR and its downstream molecule S6K1, leading to mTOR mediated cervical cancer growth and progression.^419^

##### Lung Cancer

**Fig. 4:**
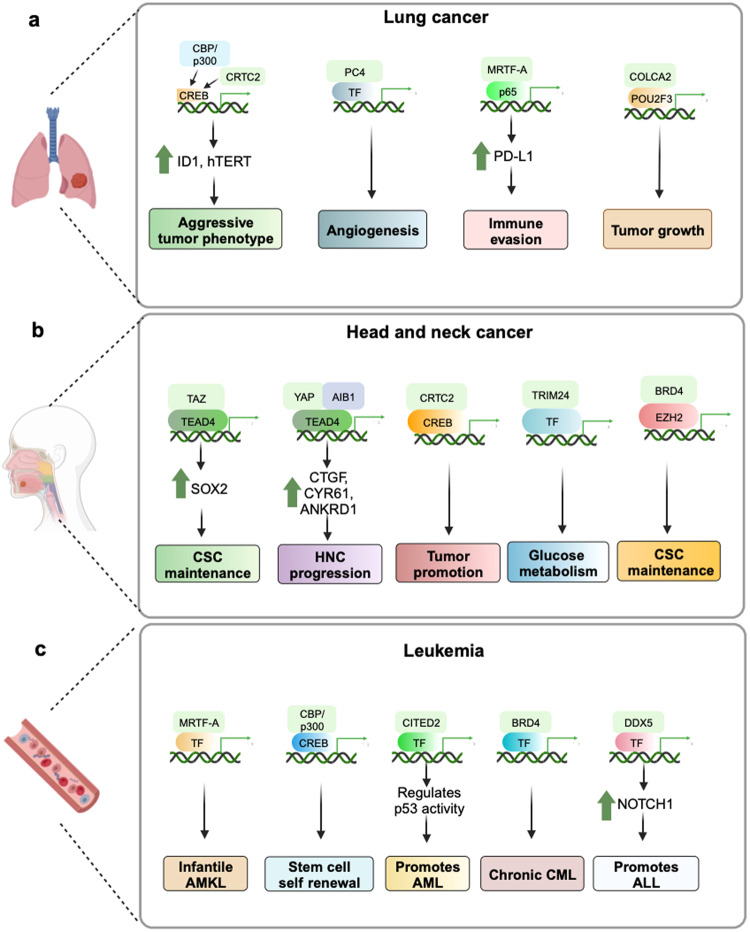
Role of transcriptional co-activators in lung cancer, head and neck cancer and leukemia. (**a**) In lung cancer, non-small cell lung cancer (NSCLC) growth is facilitated by the activity of several transcriptional co-activators including CRTC2, CBP/p300, PC4 and MRTF. The transcriptional co-activator COLCA1 interacts with the transcription factor POU2F3 to promote small cell lung cancer (SCLC) growth. (**b**) In head and neck cancers (HNC), YAP and AIB1 interact with TEAD family of transcription factors to facilitate tumorigenesis. TAZ, on the other hand, forms a complex with TEAD4, binds to the promoter region of the pluripotency gene *SOX2*, consequentially initiating its transcription, thereby upregulating self-renewal and maintenance of CSC population. The co-activator molecules CRTC2, TRIM24 and BRD4 have also been reported to be actively involved in head and neck cancer growth, proliferation and CSC maintenance. (**c**) In leukemia, deregulation of the co-activators MKL/MRTF-A, CBP/p300, CITED2, BRD4 and DDX5 have been observed. CSC, cancer stem cells; AMKL, acute megakaryoblastic leukemia; AML, acute myeloid leukemia; CML, chronic myelogenous leukemia; ALL, acute lymphocytic leukemia. This figure was created using BioRender (https://biorender.com/)

With an estimated 1.8 million deaths, lung cancer is the leading cause of cancer related deaths worldwide.^346^ In spite of numerous developments in treatment modality, the survival outcomes are discouraging. For the low- and middle-income countries, lung cancer is emerging as a serious health concern.^420^ So, identifying new therapeutic avenues to combat the disease is a prerogative. Accordingly, the following transcriptional regulators have been identified to act as masterminds modulating lung cancer at large (Fig. 4a).

#### POU2AF2 (C11orf53)/POU2AF3 (COLCA2)

POU2F3 (POU class 2 homeobox 3; also known as SKN-1a/OCT-11) is the master regulator of cell identity in the neuroendocrine^low^ variant of small cell lung cancer (SCLC).^421^ Co-immunoprecipitation assay performed by Zhou et al.^422^ revealed that the transcriptional co-activator COLCA2 (POU2AF3) and C11orf53 (POU2AF2) physically interacts with transcription factor POU2F3 to regulate tuft cell-like SCLC cell growth. Furthermore, mutation in N-terminal binding domain of COLCA2 reduced its interaction with POU2F3. In addition, ectopic expression of POU2F3 with COLCA2 in HEK293T cells activated the expression of AVIL, a POU2F3 direct target. However, when each factor was expressed individually, the expression of AVIL was not activated, indicating that physical interaction between transcriptional co-activator COLCA2 with transcription factor POU2F3 is essential to facilitate POU2F3-mediated gene transcription. Moreover, Colca2^–/–^ mice and C11orf53^–/–^ were reported to be viable. Therefore, disruption of this co-activator/transcription factor physical interaction is predicted to be a potential therapeutic strategy to selectively inhibit tuft cell-like SCLCs with minimal toxicities.^422,423^

#### CRTC2

Non-small cell lung cancer (NSCLC) accounts for about 80–85% of all lung cancers^424^ and about 20% of NSCLC report alterations in LKB1 (Liver Kinase B1).^425^ It has been reported that LKB1-deficient NSCLC (LKBC) is associated with aberrant dephosphorylation and activation of the transcriptional co-activator CRTC2. It has been reported that CRTC2 is highly expressed in lung cancer tissues compared to adjacent normal.^426^ Active CRTC2 shuttles to the nucleus, binds to CREB and stimulates their downstream signaling cascade.^427^ ID1 (Inhibitor of DNA Binding 1) is a canonical CREB target and the constitutive upregulation of CREB/CRTC2 pathway in LKBC promotes oncogenesis by enhancing the expression of ID1 which upon activation regulates the expression of genes responsible for extracellular matrix and cytoskeleton modulation, cell-cell interactions, anchorage-independent growth and lung colonization, thereby promoting a more aggressive tumor phenotype.^428^

#### CBP/p300

CREB-binding protein (CBP) and its paralog, E1A-binding protein (p300) are highly conserved transcriptional coactivator with four transactivation domains that mediate interaction with transcription factors.^429^ These co-activators contain histone acetyltransferase (HAT) activity, which enables them to acetylate various non-histone transcription-related proteins such as p53.^430^ Overexpression of CBP/p300 is considered as a poor prognosis indicator for lung cancer patients.^431^ Activation and upregulation of human telomerase reverse transcriptase (hTERT) is a hallmark of lung cancer. It has been reported that, upregulation of hTERT expression and tumor growth in lung adenocarcinoma cells is mediated by CBP, which binds to *hTERT* promoter and upregulates its transcription.^432^ Another study conducted by Zhang et al.^433^ reported that lncRNA LINC01977 interacts with SMAD3 and induces its nuclear transport. The nuclear SMAD3 interacts with CBP/p300 to regulate the transcription of ZEB1, thereby promoting malignancy of early-stage lung adenocarcinoma.^433^ Moreover, it has also been documented that p300 in association with CREB suppress lipid peroxidation by binding to the GPX4 (glutathione peroxidase 4) promoter region, which further inhibits ferroptosis in lung adenocarcinoma.^434^ Hence, overexpression of CBP corresponds to poor prognosis in lung cancers.

#### PC4

Large scale microarray data integration across 21 major cancer types identified the transcription co-activator PC4 (positive co-factor 4) amongst the 46 common cancer signatures.^435,436^ It has been reported that PC4 is highly expressed in NSCLC cells and tissues.^432^ Moreover, PC4 has also been associated with lymphatic metastasis and poor prognosis in lung adenocarcinoma.^437^ PC4 has been shown to mediate transcriptional activation of several oncogenes. Studies have indicated that in lung adenocarcinoma, PC4 functions as an upstream inducer of VEGF-C, VEGF-D, and VEGF-R3, which are necessary for promoting angiogenesis.^437^ Moreover, the downregulation of PC4 led to anti-tumorigenic effects on NSCLC cells involving induction of cancer cell death and differentiation. Thus, PC4 could be a potential therapeutic target for non-small cell lung carcinoma.^438^

#### MRTF

Du et al.^439^ reported that in NSCLC, the transcriptional co-activator MRTF-A (myocardin-related transcription factor-A), interacts with NF-κB/p65 rather than its common binding partner SRF (serum response factor). This interaction facilitates NF-κB/p65 binding to the PD-L1 promoter, thereby promoting the transcription activation and expression of PD-L1. This further potentiates immune escape of NSCLC cells. Moreover, overexpression of MRTF-A has also been reported to regulate the activity of HOTAIR promoter, thereby promoting proliferation and migration of NSCLC cells through lncRNA HOTAIR.^440^

##### Head and Neck Cancer

Head and neck cancer (HNC) is one of the most common cancers worldwide by its incidence rate and accounts for over 800,000 new cases annually.^346^ It contributes to almost 8.59% of total cancer-related deaths amongst male worldwide. HNC comprises of the cancers at various sites, including the lip, oral cavity, larynx, nasopharynx, oropharynx, hypopharynx, salivary glands, nasal and paranasal cavity. The most common sites of occurrence in HNC vary by geographic distribution, because the etiology of HNC is associated with the components of modern lifestyle like tobacco, alcohol, areca nut etc.^441^ For this reason, understanding basic molecular crosstalk in the pathogenesis of HNC is one of the most important aspects of management of this cancer (Fig. 4b).

#### YAP/TAZ

YAP and TAZ are transcriptional co-activators with various upstream signals, which are mainly regulated by the Hippo signaling pathway.^442^ Studies have reported that both YAP and TAZ are over-expressed in head and neck squamous cell carcinoma (HNSCC).^443^ In HNSCC, the WNT signaling pathway and the ERBB4 ICD (erbb2 receptor tyrosine kinase 4 intracellular domain) are known to elicit YAP activation. The active YAP and its functional paralog TAZ then migrate to the nucleus, form a transcriptional complex with their DNA-binding partner TEAD (transcriptional enhanced associate domain) and promotes transcription of YAP/TAZ target genes (*CTGF*, *CYR61*, *ANKRD1 etc*.), ultimately aiding tumorigenesis.^444^ Moreover, the transcriptional complex formed by TAZ and TEAD4 has two binding sites in *SOX2* promoter, which in turn facilitates transcription of SOX2, leading to the self-renewal property and maintenance of the cancer stem cell population in HNSCC cells. This further increases the risk of tumor recurrence and poor patient prognosis.^445^ Thus, targeting YAP/TAZ co-activators have high potential as targeted therapy for HNSCC treatment.

#### CRTCs

The family of cAMP-regulated transcriptional coactivators (CRTCs) is known to associate with the transcription factor cAMP response element–binding protein (CREB), which has proto-oncogenic properties.^446^ In HNSCCs, mitogen-activated kinase kinase1 (MEKK1) constitutively activates and overexpresses CRTC2 (cAMP-responsive element-binding protein (CREB)-regulated transcription coactivator 2) via a non-canonical MEKK1-p38 signaling axis. As a matter of fact, overexpression of CRTC2 leads to higher physical interactions between CREB and CRCT2, accentuating the CREB downstream signaling, which is essential for a several cancer-associated adaptive response like glucose metabolism, cell growth, survival, immune evasion, and the maintenance of cancer stem cells.^447^ It has also been reported that in salivary mucoepidermoid carcinoma (MEC), frequent CRTC1/3-MAML2 fusions were observed. This CRTC1-MAML2 interaction promotes salivary MEC development and maintenance.^448,449^

#### TRIM24

Transcriptional co-activator TRIM24 has been shown to drive cell cycle progression and upregulate CYCLIN D1 and p-Rb expression in HNSCC, suggesting that TRIM24 is involved in HNSCC progression through regulation of cell cycle related proteins.^450^ Cui et al.^451^ reported that TRIM24 variants were highly expressed in 56 HNSCC samples (P < 0.001). Furthermore, 54.95% (50/91) of HNSCC samples showed upregulated expression of TRIM24 by immunohistochemistry. In addition, univariate analysis indicated that high TRIM24 expression correlated with worse overall survival (P = 0 .020). Moreover, in multivariate analysis, TRIM24 was also recognized as an independent predictor of overall survival (P = 0 .030). In addition, TRIM24 was able to induce upregulation of GLUT3, a glucose transporter that further confirms the fact that TRIM24 regulates glucose metabolism, thereby promoting cancer metabolism.^450,452^

#### BRD4

Bromodomain containing 4 (BRD4) is a protein that associates with acetylated histones through its double bromodomains and facilitates transcription of the downstream genes.^453^ A significant overexpression of BRD4 in primary HNSCC samples as well as 4-nitroquinoline 1-oxide (4NQO)-induced HNSCC animal model was found to assist cell proliferation, migration, and invasion.^454^ Another study revealed that BRD4 and MMP2 expression levels were correlated in oral squamous cell carcinoma (OSCC), and both were highly expressed in lymph node metastasis cases, including delayed metastasis, and that suggests its potential use as novel predictor of metastasis.^455^ BRD4 has also been found to facilitate spheroid formation and invasion through a BRD4/EZH2 pathway which non-canonically activates STAT3 transcription factor, thereby promoting tumor progression through ΔNp63α-mediated transcription, which strongly suggests its possible involvement in cancer stem cell maintenance.^456^

##### Leukemia

Leukemia is a malignancy that is characterized by transformed hematopoietic progenitors and by bone marrow infiltration.^457^ According to Globocan 2020, leukemia accounted for 4% of total cancer-related death in males and 2.7% of total cancer-related death in females.^346^ Therefore, with the rapid increase in leukemic burden, addressing challenges in curbing the disease is imperative (Fig. 4c).

#### MKL1/MRTF-A

MKL1/MRTF-A has first been identified as a part of recurrent t (1;22) chromosomal translocation in acute megakaryoblastic leukemia. This translocation is specific to infantile AMKL and has majorly been diagnosed in patients younger than 6 months of age.^457^ Studies concerning MKL1/MRTF-A requires further investigation.

#### CBP/p300

Mixed lineage leukemia (MLL) gene has been reported to recruit p300/CBP through its transcriptional activation domain, which further promotes acetylation of histone H3 at lysines 9, 18, and 27. The AF4 family/ENL family/P-TEFb complex (AEP) binds to acetylated H3K9/18/27 to activate transcription, consequently activating the cellular machinery required for aberrant self-renewal of leukemia stem cells.^458^ CBP/p300 has also been reported to mediate the leukemic functions of MYB.^459^

#### CITED2

Elevated expression levels of the co-activator CITED2 (CBP/p300-interacting-transactivator-with-an-ED-rich-tail 2) has been associated with maintenance of both normal and leukemic hematopoietic stem and progenitor cells (HSPCs).^460^ Moreover, a subset of AML patients displayed higher expression levels of CITED2 in CD34(+) cells as compared with normal CD34(+) HSPCs.^461^ CITED2 also regulates p53 activity to promote AML, and therefore it can be a potential target for AML therapy.

#### BRD4

BRD4 has been found to be expressed in primary CML cells, CD34^+^/CD38^−^ leukemic stem cells (LSC), and in the CML cell lines KU812, K562, KCL22, and KCL22T315I.^462^ Collaboration between BRD4 and DOT1L has been reported to be important in highly transcribed genes in proximity to super enhancers. By means of dimethylated histone H3 K79, DOTL1 facilitates histone H4 acetylation, consequently regulating the binding of BRD4 to chromatin Moreover, inhibition of BRD4 activity was found to suppress proliferation in the majority of patients with chronic phase CML.^463^

#### DDX5

An important oncogenic mechanism for T cell acute lymphoblastic leukemia (T-ALL) is aberrant Notch signaling. It has been observed that the transcriptional co-activator DDX5 acts as a component of MAML1 protein complex to facilitate NOTCH1 transcription activation complex in human T-ALL leukemic cells.^464^ Human T-cell leukemia virus type 1 (HTLV-1) is a causative agent of adult T-cell leukemia/lymphoma (ATL). Moreover, DDX5 and its paralog DDX17, has been reported to promote alternative splicing of cellular genes after NF-κB activation by HTLV-1, to facilitate the initiation of leukemic state.^465^

#### Colorectal cancer

**Fig. 5:**
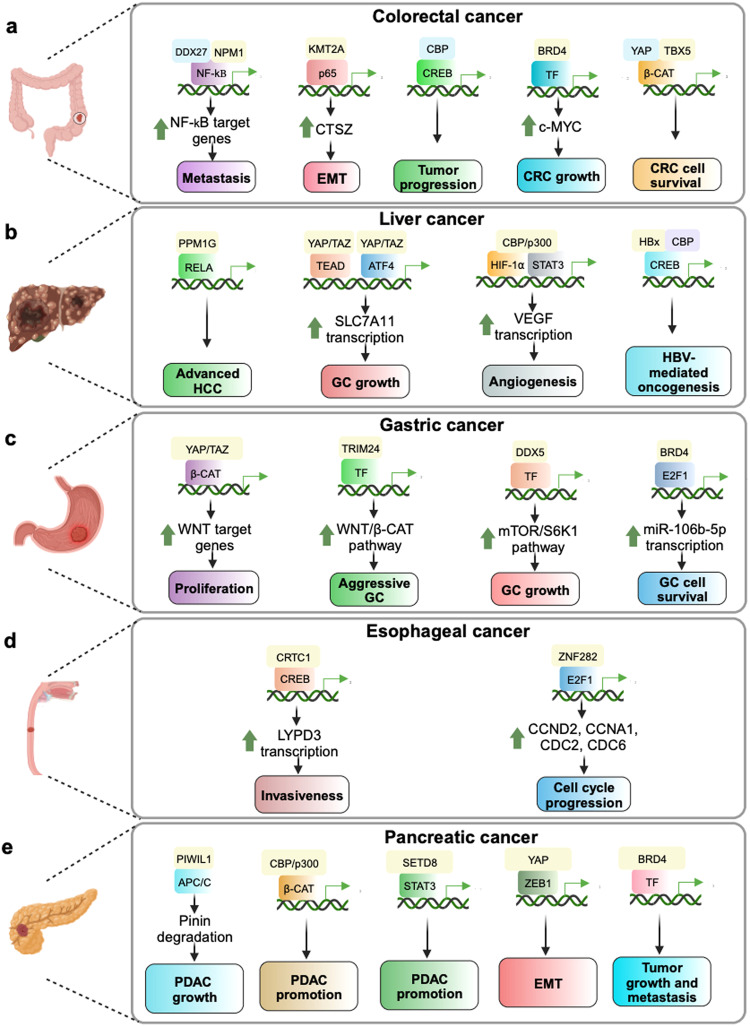
Pleiotropic influence of transcriptional co-activators in driving gastrointestinal cancers. The malignancies associated with GI tract contribute to one-third of all cancer-related deaths, with colorectal cancer, liver cancer, stomach cancer, esophageal cancer, and pancreatic cancer being the main contributors. **a** Transcriptional co-activators DDX27, KMT2A, CBP, BRD4, and YAP interacts with diverse transcription factors to facilitate colorectal cancer progression. **b** YAP, TAZ, CBP/p300, PPM1G, and HBx are significantly associated with liver cancer progression. **c** Gastric/Stomach cancer progression is modulated chiefly by YAP/TAZ and TRIM24-mediated regulation of WNT/β-CATENIN pathway, DDX5-mediated upregulation of mTOR/S6K1 pathway, and BRD4/E2F1-mediated upregulation of miR-106b-5p. **d** Transcriptional co-activators CRTC1 and ZNF282 have been reported to physically interact with transcription factors CREB and E2F1, respectively, fostering esophageal cancer growth and metastasis. **e** The co-activators PIWIL1, CBP/p300, SETD8, YAP, and BRD4 have been established to be key regulators of pancreatic cancer promotion and proliferation. PDAC pancreatic ductal carcinoma, APC/C anaphase promoting complex/cyclosome. This figure was created using BioRender (https://biorender.com/)

In the year 2020, more than 1.9 million new cases of colorectal cancer (including anus) and 935,000 deaths were estimated to occur, which represents about 10% of total cancer cases and cancer-related deaths. Overall, colorectal cancer ranks third in terms of incidence, but second in terms of mortality,^346^ and is therefore of immense concern as far as novel and specific therapeutic strategies are concerned (Fig. 5a).

#### DDX27

Chromosomal instability (CIN) is a hallmark of colorectal cancer, which results in copy number alterations (CNAs).^466^ DDX27, transcriptional co-activator is significantly upregulated and has extremely high frequency of copy number gain in colorectal cancer (CRC), and has also been found to be upregulated in CRC tissues.^467^ A study by Tang et al.^468^ has identified NF-κB pathway as the principal target of DDX27 in CRC. DDX27 binds with NPM1 (nucleophosmin1) to interact with NF-κB in the nucleus leading to increased binding of NF-κB to the target gene promoters, triggering enhanced expression of VIMENTIN and SLUG, thereby promoting metastasis in CRC. Thus, in the context of colorectal cancer, the functional interaction between DDX27-NPM1-NF-κB is essential for tumor progression and metastasis.^468^

#### CBP/p300

The transcriptional co-activators CBP (CREB-binding protein) and p300 are histone acetyltransferases (HATs) that regulate tumor initiation and progression. It has been observed that prolonged poor prognosis was associated with high expression of CBP/p300. This finding indicated that CBP/p300 could be a potential therapeutic target for CRC treatment.^469^ Xu et al.^470^ further reported that mesenchymal stem cells in the tumor microenvironment secretes CCL7. The CCL7/CCR1 in turn activates CBP/p300, which upon activation acetylates KLF5 and promotes CRC proliferation and metastasis.

#### KMT2A

The KMT family of histone modification enzymes contain the SET domain that regulates gene transcription by promoting methylation of H3K4. KMT2A/KMT2D, KMT2C/KMT2B, SETd1A/SETd1B are the three pairs of KMT members that play significant role in tumorigenesis.^471^ KMT2 family mutations has been positively correlated with CRC progression.^472^ It has been documented that KMT2A interacts with p65 transcription factor (p65 is also known as nuclear factor NF-kappa-B), which is essential for its recruitment on the promoter region of *CATHEPSIN Z* (*CTSZ*), which is one of the important downstream targets of KMT2A. Upon recruitment on the promoter, KMT2A trimethylates H3K4, that in turn promotes *CTSZ* transcriptional activation, leading to enhanced epithelial-to-mesenchymal transition in CRC cells.^473^

#### BRD4

Bromodomain-containing protein 4 (BRD4) mediates its role as transcriptional co-activator by acting both as a passive scaffold to promote recruitment of transcription factors and as an active kinase to phosphorylate RNA polymerase II, thereby regulating transcription.^474^ Upregulated expression of transcriptional regulator BRD4 is frequently observed in CRC. Targeting BRD4 resulted in significant downregulation in the expression of *MYC* proto-oncogene, restraining colon cancer progression.^475^ Wang et al.^476^ observed that in CRC, BRD4 phosphorylation has been reported to promote interaction with STAT3 to subsequently induce chromatin remodeling through enhanced binding interactions with enhancers and super-enhancers, thereby supporting a tumor-promoting transcriptional program. Moreover, it has also been reported that upon loss of mediator kinase, MED12 and BRD4 cooperate to sustain colorectal cancer growth.^477^

#### YAP1

In sporadic CRC, a SNP located in the YAP1 gene has been identified as a common genetic risk variant with a hazard ratio of 1.05 and over expression of YAP1 is associated with shorter survival.^478^ It has been documented in β-CATENIN-driven colorectal cancer, that YES1 (a tyrosine kinase) phosphorylates YAP1 on Y357, subsequently promoting YAP1 nuclear localization and activation. The active YAP1 transcriptional co-activator then interacts and forms a ternary complex with the transcription factor TBX5 and β-CATENIN to promote CRC survival and progression.^479^ Furthermore, YAP/TAZ-TEAD4 complex has been documented to transcriptionally upregulate the expression of CCBE1 (collagen and calcium-binding EGF domain 1) by binding to CCBE1 enhancer region of both CRC cells and cancer-associated fibroblasts. This in turn upregulates VEGFC proteolysis and induces lymphangiogenesis in a CRC cell-derived xenograft model in vivo.^480^

##### Liver cancer

In 2020, an estimated 830,200 people died from liver cancer globally. Global age-standardized mortality for liver cancer was 8.7 per 100,000 people and was highest in the eastern part of Asia.^481^ Hepatocarcinogenesis involves the synergistic action of several cellular mechanisms including the transcription of several factors associated with inflammation, oxidative stress, hypoxia, along with other molecular mechanisms.^482^ Therefore, a proper understanding of the mechano-molecular aspects of hepatocarcinogenesis and identification of appropriate target molecules and signaling pathways responsible for tumor progression is crucial in order to develop effective therapies against hepatic cancers (Fig. 5b).

#### PPM1G

The most commonly identified coactivators that are associated with modification of epigenetic landscape are histone acetyltransferases (HATs), deacetylases (HDACs), kinases, and phosphatases.^483^ PPM1G/PP2Cγ phosphatase (one member of a family of metal-dependent Ser/Thr phosphatases) has been identified as a NF-κB transcriptional coactivator. This particular co-activator mediates its function by binding to the NF-κB target gene promoters in association with the RELA subunit of the NF-κB family, thereby facilitating the transition between initiation and elongation.^484^ Intriguingly, high levels of PPM1G were noted in advanced hepatocellular carcinoma stages. Further experimentation revealed that in hepatocellular carcinoma, MYC/MAX and EP300 activate PPM1G which in turn dephosphorylates SRSF3, triggering the alternative splicing of genes related to cell cycle and transcriptional regulation.^485^

#### YAP/TAZ

YAP and TAZ have been identified as key players associated with Sorafenib resistance in hepatocellular carcinoma (HCC). In a TEAD- and ATF4-dependent manner, YAP/TAZ enables HCC cells to overcome Sorafenib-induced ferroptosis. Mechanistically, in a TEAD-dependent manner, YAP/TAZ induces the expression of SLC7A11, a key transporter maintaining intracellular glutathione homeostasis. At the same time, YAP/TAZ sustains protein stability, nuclear localization, and transcriptional activity of ATF4, which in turn cooperatively induce SLC7A11 expression.^486^ It has also been reported, upon MYC/β-CATENIN activation, YAP/TAZ accumulated in HCC cells to promote mitogenic activation, tumor growth and survival.^487^ Another recent study has further highlighted that TAZ is a direct transcriptional target of c-MYC, which further promotes c-MYC-driven murine hepatocarcinogenesis by regulating the expression of the anti-apoptotic BCL2L12 gene.^488^ Moreover, pertaining to ubiquitous activation of YAP and TAZ in human liver cancers,^489^ YAP/TAZ-based rewiring strategies can be potential approaches to overcome HCC therapy resistance.

#### CBP/p300

The transcriptional co-activators CBP/p300 mediates increased acetylation of H3K18 and H3K27 in HCC tissues.^490^ It has been reported that the transcription factors HIF-1α and STAT3 can maximally induce transcription of VEGF when in association with CBP/p300 co-activator. Moreover, interruption of this transcriptional complex by melatonin prevented HIF-1α occupancy of the *VEGF* promoter and prevented HCC progression. Thus, administering pharmacological doses of melatonin, a well-known dietary supplement, may be highly beneficial in inhibiting liver cancer by disrupting the HIF-1α/STAT3/CBP/p300 complex.^491^

#### HBx

Hepatitis B virus X protein (HBx) has been found to be overexpressed in liver cancer tissues.^492^ It acts as a transcriptional co-activator through direct interaction with various proteins, such as the TATA-binding protein, RPB5 subunit of RNA polymerase II, TF IIH, TF IIB, and the proteins of basic domain-leucine zipper (bZIP) family, including the cyclic AMP-response element (CRE)-binding protein (CREB).^493^ HBx interacts with the co-activators CBP/p300 and cooperates with CBP/p300 in the CREB-mediated activation. Thus, HBx may be considered as a potentiator of the signal mediated by CREB, and this mechanism may be involved in HBV-mediated oncogenesis.^494^ HBx has also been found to act as co-activator of heat shock factor 1 (HSF1) to upregulate the expression of HSPA8 in liver cancer cells. HSPA8 upon expression, enhanced HBV replication and dampened ferroptosis-mediated cell death by upregulating the expression of SLC7A11/GPX4 and by decreasing the erastin-mediated ROS (reactive oxygen species) and Fe^2+^ accumulation in cells, thereby supporting liver cancer progression.^495^ Moreover, HBx mediates H3K4me3 modification in WDR5-dependent manner^496^ and it also has been documented to interact with MYH9, to induce its expression by modulating GSK3β/β-catenin/c-Jun signaling.^497^ Futhermore, HBx interacts with ARRB1 and the autophagic core protein MAP1LC3/LC3 to induce ARRB1-mediated autophagy. This autophagic induction drives G1/S cycle and promotes HCC.^498^

##### Gastric cancer

Gastric cancer remains an important cancer worldwide as it was responsible for over one million new cases and an estimated 769,000 deaths in 2020. Incidence rates are twofold higher in males than in females. In males, it is one of the most commonly diagnosed cancers and the leading cause of cancer-related deaths in several Asian countries^346^ (Fig. 5c).

#### YAP/TAZ

One of the main risk factor for gastric cancer is *Helicobacter pylori* infection. It has been reported that deregulation of the Hippo pathway in the gastrointestinal tissues is one of the prime causes of *H. pylori*-mediated gastric carcinogenesis. Upon *H. pylori* infection, an increase in both TAZ nuclear expression and transcriptional activity of transcriptional enhancer TEA domain (TEAD) transcription factors was observed, which in turn induced EMT, invasion, and cancer stem cell-like properties.^499^ It has also been observed that a ubiquitously expressed protein tyrosine phosphatase, SHP2 (Src homology-2 domain-containing protein tyrosine phosphatase-2) interacts with the transcriptional co-activators YAP/TAZ, which in turn promotes its nuclear localization. In the nucleus, SHP2 mediates parafibromin/β-catenin complex formation, stimulating WNT-target gene activation.^500^ Based on the gene ontology (GO) analysis, it was determined that blood microparticle, platelet alpha granule lumen, and chylomicron are common cellular locations of YAP and TAZ. However, a functional divergence between YAP and TAZ was perceived owing to the GO terms focal adhesion (FA) and cell-substrate junction, which were particularly enriched in YAP-targets, suggesting that in gastric cancer cells, YAP plays a crucial role in regulating cell-substrate junctions.^501^

#### TRIM 24

Gastric cancer cell lines and tissues frequently manifest abnormally upregulated expression of TRIM-24 transcriptional co-activator. A study has reported that downregulated expression of miR-511 is essential for sustained expression of TRIM24.^502^ TRIM24, when active, promotes cell proliferation, migration and invasion by activating the WNT/β-CATENIN signaling pathway.^503^

#### BRD4

Epigenetic regulation requires the involvement of three different types of proteins. First are the enzymes that modify histones or DNA, and are known as writers. Second are enzymes that remove modifications on histone or DNA, the erasers, and third are the proteins that recognize these modifications, known as readers.^504^ BRD4, the bromodomain containing transcriptional co-activators belong to the class of epigenetic readers. It has been reported that the expression of BRD4 in human GC tissues correlates with shortened metastasis-free gastric cancer patient survival.^505^ It has been observed that BRD4 associates with the transcription factor E2F1 via its two bromodomains. This association promotes the recruitment of BRD4 to the promoter of *miR-106b-5p*, thereby facilitating its transcription. An active miR-106b-5p targets 3′-UTR of p21, eventually to regulate cellular senescence.^506^ Qin et al.^507^ further reported that the epigenetic reader BRD4 recognizes acetylated lysine 146 (K146) and K187 on Snail. This prevents Snail recognition by its E3 ubiquitin ligases FBXL14 and β-Trcp1, consequently promoting metastasis by inhibiting Snail polyubiquitination and proteasomal degradation.

#### DDX5

DDX5, DEAD (Asp-Glu-Ala-Asp) box helicase 5 is a transcriptional co-activator that is overexpressed in different malignancies and associated with progression of cancer.^508^ It has been reported that in gastric cancer tissues, DDX5 is dramatically upregulated, and its overexpression correlates with gastric cancer cell growth and invasion.^509^ It has also been observed that DDX5 promotes cell proliferation by upregulating mTOR/S6K1 signaling activity, stipulating that targeting DDX5/mTOR/S6K1 might be a novel therapeutic approach for the treatment of gastric cancer.^510^

#### MRTF

The transcriptional co-activator MRTF-A upregulates the expression of miR-155 promoter by inducing histone acetylation and RNA polymerase II recruitment. Subsequently, miR-155 suppresses the expression of SOX1 to promote gastric cancer cell migration.^511^ Furthermore, Wang et al.^512^ showed that MICALC2-mediated upregulation of nuclear MRTF-A promotes CDC42 activation, MMP9 expression, and gastric cancer cell migration.^512^

##### Esophageal cancer

Approximately 604,000 new cases of esophageal cancer have been reported with almost 544,000 deaths only in the year 2020. Esophageal cancer is responsible for one in every 18 cancer-related deaths in 2020. Approximately 70% of cases occur in men, and there is a twofold to threefold difference in incidence and mortality rates between the sexes. It is responsible for 8.3% of cancer-related deaths in males throughout the world^346^ (Fig. 5d).

#### CRTCs

Liver kinase B1 (LKB1) is an essential serine/threonine kinase that is downregulated in a subset of esophageal tumor. Owing to this downregulation, LKB1 is unable to downregulate the expression of CREB-regulated transcription co-activator 1 (CRTC1), leading to their aberrant activation. Mechanistically, upon activation, CRTC1 interacts with the CREB transcription factor and enhances the expression of CREB target genes like *LYPD3*, a high-glycosylated cell surface protein,^513^ ultimately augmenting cell migration and invasion, which contributes to esophageal cancer progression.^514^ On the other hand, CRTC2 in cooperation with CBP/p300 deposits acetylation marks on histones at inflammatory gene loci, consequently promoting active transcription and cytokine expression. This CRTC2-CBP/p300-mediated histone modification, links metabolic and epigenetic states to inflammatory potential in esophageal cancer.^515^

#### ZNF282

E2F1 transcription factor is a key player that modulates cell cycle, DNA damage response, and apoptosis.^516^ It has been observed that ZNF282 (Zinc finger protein 282) functions as an E2F1 co-activator in esophageal squamous-cell carcinoma (ESCC), inducing accelerated transcription of E2F1 target genes like *CCND2*, *CCNA1*, *CDC2*, and *CDC6*, facilitating G1/S transition and cell cycle progression. Moreover, in comparison to normal esophageal epithelium, ZNF282 has been frequently reported to be overexpressed in ESCC tissues and ZNF282 depletion increased apoptosis and promoted cell cycle arrest at G1/S, suggesting that ZNF282 transcriptional co-activator, plays pivotal role in controlling E2F1-mediated ESCC progression.^517^

##### Pancreatic cancer

According to Globocan (2020), pancreatic cancer accounts for 5.3% and 3.8% of total cancer-related deaths in males and females, respectively.^346^ The most common concern of pancreatic cancer is the detection of the disease at advanced stages as the patients seldom exhibit symptoms at the earlier stages. Alarmingly, as sufficient causes of pancreatic cancer have not been deciphered yet, therefore identifying potential therapeutic targets might assist in the abrogation of this disease^518^ (Fig. 5e).

#### PIWIL1

Piwi-like protein 1 (PIWIL1) is encoded by the PIWIL1 gene in humans and the expression of this gene is generally restricted to germ cells. Li et al.^519^ have shown that human PIWIL1 in apo state (without piRNA binding), acts as a co-activator of anaphase promoting complex/cyclosome (APC/C) E3 complex, which in turn selectively targets a cell adhesion-related protein, Pinin, for degradation and enhances pancreatic ductal carcinoma (PDAC) metastasis. Moreover, at mRNA and protein levels, the expression of PIWIL1 was found to be associated with progenitor molecular subtype of pancreatic cancer, indicating that in resectable pancreatic cancer, PIWIL1 can be considered as a potential prognostic marker.^520^

#### CBP/p300

CBP/p300 is highly expressed in pancreatic tissues in comparison to normal tissues. Manegold et al.^521^ demonstrated that when CBP is active, it acts as a co-activator of β-CATENIN and induces PDAC progression. On the contrary, upon pharmacological inhibition of CBP, its homologous co-activator p300 interacts with β-CATENIN to promote differentiation and renders the cancer cells susceptible to therapy. Inhibition of p300 by XP-524 has also been reported to increase oncogenic KRAS, which is found to be expressed in 90% of the PDAC cases. This indicates that p300 might play an important role in PDAC progression.^522^

#### SETD8

The methyl transferase SETD8 has been documented to be upregulated in pancreatic cancer. Liu et al.^523^ have reported that SETD8 interacts with STAT3 and induces monomethylation of H4K20 on DUSP10 promoter, thereby promoting epigenetic silencing of DUSP10 (Dual Specificity Phosphatase 10). Inhibition of DUSP10, consequently promotes the upregulation of ERK1/2, consequently promoting pancreatic adenocarcinoma. It was observed that SETD8 interacts with promoter region of RRAD to reduce the levels of lipid peroxidation, which further inhibits ferroptosis-mediated death of pancreatic cancer cells.^524^

#### YAP

A recent study by Zhou et al.^525^ has documented that in comparison to normal controls, YAP1 is the most highly expressed protein in pancreatic cancer tissues (log2 fold change 6.4; *p* = 5E−06). Moreover, YAP has also been demonstrated as an independent prognostic marker in pancreatic cancer (hazard ratio 1.870, 95% confidence interval (CI) 1.224–2.855, *p* = 0.004). Liu et al.^526^ reported that YAP as a transcriptional co-activator interacts with ZEB1 to promote transcription of ITGA3, consequently enhancing EMT plasticity and spheroid formation. Furthermore, Unc-51 like kinase 1 (ULK1) interacts with YAP in the nucleus and promotes its phosphorylation-mediated stabilization. Upon stabilization, YAP facilitates PKM2 (pyruvate kinase M2) transcription, glycolysis, and PDAC cell proliferation and growth.^527^ Owing to its diverse regulatory role in pancreatic cancer, YAP can be a potential therapeutic target.

#### BRD4

Jiao et al.^528^ reported that in comparison to the adjacent non-cancerous tissues, elevated expression of BRD4 is observed in pancreatic cancer. It was also observed that BRD4 interacted with the promoter region of CAVEOLIN-2, subsequently promoting transcriptional activation of CAVEOLIN-2. Clinical studies further indicated that in pancreatic cancer patients, BRD4 (high)/caveolin-2 (high) was associated with shorter disease-free survival. Another study by Yamazaki et al.^529^ further reported that YAP/BRD4 binding at the enhancer region is associated with transcriptional activation of receptor tyrosine kinase-like orphan receptor 1 (ROR1), thereby promoting tumor growth and metastasis.

This study so far has highlighted the transcriptional co-activators that have been depicted to be deregulated across most prevalent cancer types. However, considerate inspection also reveals an involvement of common co-activators across several cancer types. Therefore, profound understanding of the molecular mechanisms by which these master transcriptional regulators exert their function will potentiate the development of a pan-disease therapeutic regime. Table 2 attempts to collate the predominance of frequently deregulated co-activators across the deadliest forms of cancer.

**Table 2:** Transcriptional co-activators across the deadliest cancer types

No.	Transcriptional co-activators	Cancer type	Reference No.
1.	ADA3	Breast cancer	^371^
2.	ARA70	Prostate cancer	^396,397^
3.	BCL9/BCL9L	Breast cancer, Prostate cancer	^354,355,393^
4.	BRD4	Ovarian cancer, Head and neck cancer, Leukemia, Colorectal cancer, Gastric cancer, Pancreatic cancer	^407,454,463,475,505,528^
5.	CBP/p300	Prostate cancer, Ovarian cancer, Lung cancer, Leukemia, Colorectal cancer, Liver cancer, Pancreatic cancer,	^386,403,431,459,469,490,521^
6.	CITED2	Breast cancer, Prostate cancer, Leukemia	^360,391,460^
7.	COLCA2	Lung cancer	^422,423^
8.	CRTC1	Head and neck cancer, Esophageal cancer	^448,514^
9.	CRTC2	Ovarian cancer, Lung cancer, Head and neck cancer, Esophageal cancer	^406,426,447,515^
10.	DDX17	Breast cancer	^369^
11.	DDX27	Colorectal cancer	^467^
12.	DDX5	Leukemia, Gastric cancer	^465,509^
13.	EYA2	Breast cancer	^373,375^
14.	HBx	Liver cancer	^492^
15.	KMT2A	Cervical cancer, Colorectal cancer	^416,472^
16.	MRTF	Breast cancer, Lung cancer, Leukemia, Gastric cancer	^350,439,457,512^
17.	PC4	Prostate cancer, Lung cancer	^394,437^
18.	PIWIL1	Pancreatic cancer	^520^
19.	PPM1G	Liver cancer	^485^
20.	SET7	Breast cancer	^364^
21.	SETD8	Pancreatic cancer	^524^
22.	SRC	Breast cancer	^358^
23.	TRIM24	Breast cancer, Ovarian cancer, Cervical cancer, Head and neck cancer, Gastric cancer,	^378,409,417,451,502^
24.	TRIM28	Cervical cancer	^419^
25.	VGLL1	Cervical cancer	^413^
26.	YAP/TAZ	Breast cancer, Ovarian cancer, Head and neck cancer, Colorectal cancer, Liver cancer, Gastric cancer, Pancreatic cancer,	^382,383,400,443,478,486,499,525^
27.	ZNF282	Esophageal cancer	^517^

#### Co-activator involvement in cancer stemness

Though multitudinal therapeutic interventions for treating cancer are used worldwide, high rate of metastasis, recurrence, and relevant mortality still persists.^530^ One of the major role-players in this are the small population of pluripotent cells residing within the tumor, known as cancer stem cells (CSCs).^531^ Within the tumor, the cancer cells and CSCs remain in a dynamic equilibrium state which is maintained by two opposing phenomena, differentiation and de-differentiation.^532^ Upon receiving certain cues, cancer stem cells can differentiate to give rise to the cancer cells or cancer cells can de-differentiate back to their CSC state.^533^ CSCs accomplish this dynamic state by re-wiring their transcriptional machinery which further determines the aggressiveness and recurrence rate of the cancer.^534^ Researchers have shown the involvement of co-activators in cancer stem cell self-renewal and maintenance. Some of these findings are discussed below.

Using a mouse model of glioma, Pietras et al.^535^ experimentally proved that Osteopontin-CD44 signaling facilitates the maintenance of the CSCs phenotypes via CBP/p300-dependent enhancement of HIF-2α activity. The CBP/p300-interacting transactivator with ED-rich tail 2 (CITED2) further suppresses the CSC markers and reduces the cancer stem cell population in NSCLC.^536^ Integrated transcriptome and protein-protein interaction studies revealed that the arginine methyltransferase PRMT6 can regulate stemness properties via MEK/ERK pathway in hepatocellular carcinoma.^537^ Zhu et al.^538^ performed Spearman correlation test using TCGA pan cancer data and found the KMT2 family genes to be associated with cancer stemness and drug sensitivity. Recently, in intestinal tumorigenesis, co-activator MLL1 was observed to govern WNT/β-Catenin induced cancer stemness.^539^ KDM2A in breast cancer enhances stemness and angiogenesis by Jagged1 (JAG1) dependent mechanism.^540^ Another lysine demethylase KDM6B has been reported to enhance stemness related genes like SOX2, SOX9, and OCT4.^541^ Li et al.^542^ identified JMJD3 as one of the main drivers of esophageal squamous cell carcinoma pathogenesis through JMJD3/MYC/miR-17–92 pathway and regulate stemness and sensitivity to therapy. Steroid receptor co-activator 1 and 3 (SRC1/3) plays a crucial role in CSC state maintenance and metastasis in breast cancer cell lines. In addition, it was also observed that siRNA mediated knockdown of SRC1/3 significantly reduced the CSC population.^543^ Jaworska et al.^544^ in their study have summarized the role of different proteins of the TRIM co-activator family in modulating different signaling pathways associated with self-renewal of CSCs. One more study found TRIM29 stabilizes interferon-stimulated gene 15 (ISG15) and promote cancer stem cell-like phenotype in pancreatic ductal adenocarcinomas (PDACs).^545^ Recent findings have suggested the involvement of YAP/TAZ in stemness maintenance and their deregulation may induce transformation of the cancer cells into CSCs.^546^ It is now quite evident that the co-activators not only take part in tumor growth but are also responsible for cancer stem cell fueled metastasis, drug resistance and unresponsiveness to therapies. Oncologists are still in search of therapies to abrogate both the cancer stem cell population along with the differentiated cancer cell population. Due to their active collaboration in almost all aspects of cancer progression, the possibility of co-activator-based CSC targeting strategies in successfully eliminating both the CSC and non-CSC components of cancer, will retrench tumor relapse or secondary tumorigenesis.

## Applications in biomedical research and targeted therapy

### Importance of co-activators in disease research and drug discovery

**Fig. 6:**
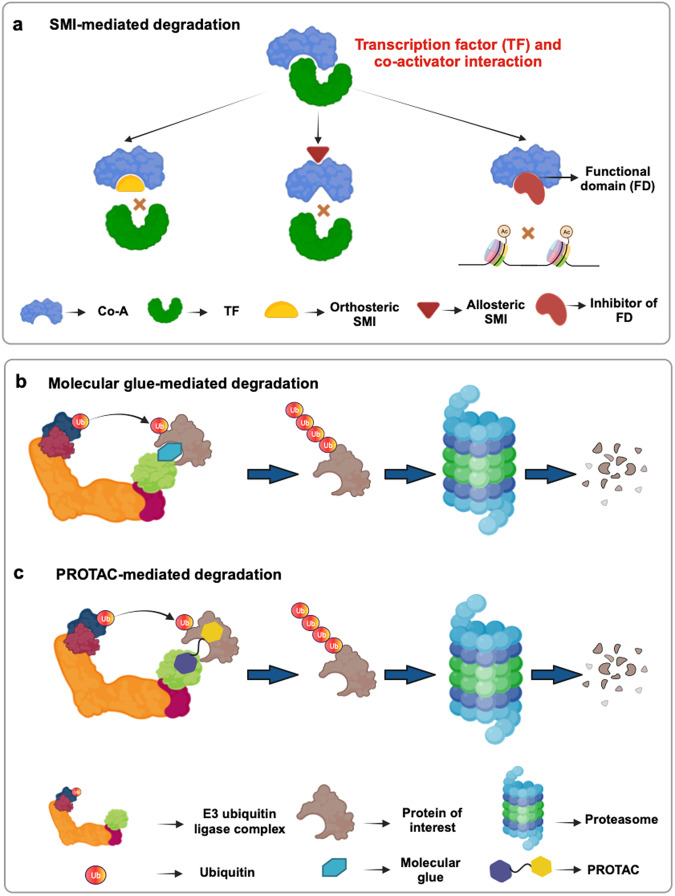
Current therapeutic strategies to target transcriptional co-activators. **a** Targeting the co-activators (CoA) with small molecule inhibitors (SMIs) is a widely used strategy to inhibit the function of the CoAs. The SMI interacts with binding residues on the target protein surface and mediates either orthosteric or allosteric inhibition. During orthosteric inhibition, SMIs directly block protein-protein interactions with their binding partners. Allosteric inhibition is achieved when the SMIs bind to the target proteins and induce conformational changes on the binding surface, thereby dampening its activity. SMIs have also been reported to bind to functional domain (FD), like the bromodomains, of the transcriptional CoAs, consequently preventing the interaction between the acetyl groups and the bromodomains to inhibit transcriptional activation. **b** Molecular glue degraders are another potential therapeutic strategy. The interaction between an E3 ubiquitin ligase and the transcriptional co-activators are induced by the molecular glue, which promotes ubiquitination-mediated degradation of the co-activators. **c** The most rapidly growing heterobifunctional protein degrading system is proteolysis-targeting chimeras (PROTACs). The degradation system of PROTACs comprises an anchor and a warhead, which is connected by a linker molecule. The warhead binds to the protein of interest (POI), while the anchor recruits E3 ubiquitin ligase, thereby hijacking the ubiquitin proteasome system of the cell to degrade the POI. This figure was created using BioRender (https://biorender.com/)

TFs, in order to swiftly integrate cellular stimuli, must have the ability to rapidly recruit multiple proteins associated with transcription machinery using single short domains. By the course of evolution, TFs have adapted to perform such function by promptly producing an ensemble of malleable structures that are modified depending on its binding partners.^1,6^ As a consequence, they do not possess specific structural integrity, making it difficult to target them. Researchers have attempted to device alternative targeting strategies, specifically involving the transcriptional co-activators. It is well-established that co-activators are modulated by signal transduction, and depending on the received information, they drive TF activity in the context of gene expression.^16,17^ Hence, development of strategies to modulate the co-activators is worth exploring since they can be easily and safely targeted, and can effectively alter the function of the TFs. Such ideas have now been practically implemented in the field of drug discovery and multiple new strategies such as small molecule inhibitors (SMIs), proteolysis-targeting chimeras (PROTACs) and molecular glue degraders targeting the co-activators are being considered through extensive research (Fig. 6).

Previously, in this review, involvement of co-activators in different mechanomolecular aspects of transcriptional regulation and their association with multiple disease phenotypes have been elaborated. Numerous studies have also identified co-activator gene mutations as drivers of multiple diseases (Table 1). Altogether, these findings suggest that targeting the co-activators will not only reduce the transcription factor-mediated malicious gene expression, but will also disrupt the molecular interconnections between multiple disease-causing pathways, leading to better patient prognosis.

### Small molecule inhibitors of transcriptional co-activators

Small molecule inhibitors (SMIs) are the chemical compounds having the molecular weight of <500 Da and they have been reported to interact with the binding pockets present on the surface of the target proteins to disrupt their functionality^547^ (Fig. 6a). Use of SMIs has gained wide popularity owing to several selective advantages. First, the SMIs can be easily prepared and structurally modified based on conceptually straightforward techniques. New compounds with greater potency can be readily generated by modulating the structural conformation of the old compounds. Moreover, due to the well-known chemical groups in their molecular composition, SMIs with high penetration property and rapid metabolism can be created effortlessly.^547,548^ Therefore, treatment with SMIs is a lucrative therapeutic strategy to target diseases associated with deregulated co-activator function. The selective SMIs of transcriptional co-activators across different disease phenotypes, along with their mechanism of action, have been summarized in Table 3.

**Table 3:** Transcriptional co-activators and their small molecule inhibitors: pathological perspective

Co-activator	Small molecule inhibitor	Disease/Model of investigation	Mechanism of inhibitor action	Ref.
YAP/TAZ	Cerivastatin	NSCLC	The mevalonate pathway-associated rate limiting enzyme inhibitor Cerivastatin, promoted cytoplasmic retention and subsequent degradation of YAP	^627^
	Dasatinib	TNBC	SRC kinase activity is essential for YAP/TAZ activation. Dastinib inhibits SRC kinase which further inhibited nuclear localization of YAP/TAZ and decreased YAP/TAZ-TEAD-dependent reporter activity	^628^
	Fluvastatin	TNBC	Fluvostatin-mediated inhibition of HMG-CoA reductase leads to YAP/TAZ inactivation and halted nuclear translocation through disrupted geranylgeranylation of RhoA	^629^
	DC-TEADin02	HEK293T and HCT 116 cells	Palmitoylation of TEAD is important for stability and interaction with YAP/TAZ. DC-TEADin02 is a vinylsulfonamide derivative and a covalent TEAD autopalmitoylation inhibitor	^630,631^
	Fenamate	Glioblastoma	Use of a chloromethyl ketone substitution to link fenamate with TEAD disrupts the YAP-TEAD interaction	^632^
	Alkylthio-triazole scaffold	HeLa, JHH7 and HuH7 cell lines	These compounds occupy palmitate-binding pocket and prevent interaction between YAP/TAZ and TEADs	^633^
	K-975	Malignant pleural mesothelioma	Binds to Cys359 in PBP via an acrylamide structure to inhibit YAP/TAZ-TEAD interaction	^634^
	TEAD destabilizers	Not specified	Bind to PBP to destabilize TEAD conformation through in situ unfolding thereby preventing YAP/TAZ-TEAD interaction	^635^
	MSC-4106	NCI-H226 tumor xenograft model	Prevents TEAD1/ TEAD3 auto-palmitoylation to inhibit activity of YAP/TAZ	^636^
	Verteporfin (VP)	NSCLC	Increases erlotinib sensitivity in H1975 cells. In combination with erlotinib, VP reduced invasion, migration and sphere-forming ability.	^637^
	Verteporfin (VP)	Liver cancer	Verteporfin-mediated inhibition of YAP/TAZ signaling significantly improved transcatheter arterial chemoembolization in transplanted hepatocellular carcinoma (HCC)	^638^
	Verteporfin (VP)	Gastric cancer	VP disrupts YAP/TAZ-TEAD interaction to decrease the pool of gastric cancer stem cells.	^639^
	Verteporfin (VP)	Head and neck cancer (HNSCC)	VP suppresses proliferation and metastasis by inhibiting YAP1. Verteporfin in combination with melatonin was also found to suppress survival and maintenance of head and neck cancer stem cells.	^640,641^
	Verteporfin (VP)	Breast cancer	Independent of light activation, VP mediated Caspase-9 cleavage and PARP cleavage induced apoptosis in the cells of various breast cancer subtypes by inhibiting YAP. Nano-encapsulated verteporfin in combination with combretastatin and paclitaxel inhibited breast cancer stem cells, bulk cancer cells, and angiogenesis.	^642,643^
	Pazopanib	TNBC	Pazopanib inhibits RHOA activity by inhibiting VEGFR and PDGFR, thereby promoting inhibitory phosphorylation of YAP/TAZ and their subsequent proteosomal degradation	^644^
	GNE-7883	YAP/TAZ-dependent cancer cells like MDA-MB-231 NCI-H226, Detroit 562, HCC1576	Mediates allosteric inhibition of YAP/TAZ-TEAD interaction by binding to TEAD lipid pocket	^645^
	YAP/TAZ inhibitor-1	HER2-Positive Breast Cancer	Inhibits YAP/TAZ to reverse Trastuzumab resistance	^646^
	Atorvastatin and zoledronic acid (YAPPETIZER)	TNBC	Inhibits YAP/TAZ expression via mevalonate pathway (Phase II clinical trials; NCT03358017)	^647^
	HDAC inhibitors (Entinostat)+ Molibresib besylate (BET inhibitor)	Advanced and refractory solid tumors and lymphomas	This drug combination is in Phase I clinical trial (NCT03925428) and its exploratory objective includes checking of YAP activity after drug treatment	^648^
	Benzisothiazole-dioxide scaffold	Malignant pleural mesothelioma, lung cancer, breast cancer	Disrupts YAP/TEAD interaction by binding to TEAD surface	^649^
	VT103, VT104, VT105, VT106, and VT107	Mesothelioma cell lines	Inhibit TEAD auto-palmitoylation	^650^
	Cytochalasin-D	Melanoma	Inhibits actin polymerization and promotes cytoplasmic retention of YAP/TAZ	^651^
BET	JQ1	TNBC	TNBC cell lines show growth inhibition when treated with JQ1	^652^
	JQ1	Colorectal cancer (CRC)	JQ1 synergized with PD-1 blockade and enhanced anti-tumor activity by remodeling the immunosuppressive niche	^653^
	JQ1	SCLC	Blocks NEUROD1 transactivation	^654^
	OTX-015/JQ1	Liver cancer	Both the BET inhibitors downregulated HCC migration by inhibiting SMARCA4	^655^
	JQ1	Esophageal cancer	JQ1 prevents YAP1 activity by abolishing the interaction of BRD4 with YAP1 promoter	^656^
	JQ1	Stomach cancer	JQ1 inhibits RUNX2/NID1 signaling to inhibit gastric cancer progression	^657^
	JQ1	Cervical cancer	JQ1 inhibits BRD4 to sensitize cervical cancer cells to radiotherapy and inhibits the Plk1-Mutant Trp53 Axis.	^658^
	JQ1	HNSCC	Therapeutic targeting of BRD4 is a potent anti-cancer strategy.	^659^
	ABBV-075	HCC	Inhibit proliferation and migration of HCC	^660^
	BET-IN-8 (Compound 27)	Sepsis	BET-IN-8 (2-((2-methylbenzyl) thio)-6-oxo-4-(3,4,5-trimethoxyphenyl)-1,6-dihydropyrimidine-5-carbonitrile) is an effective bromodomain inhibitor of BRD4 which reduces pro-inflammatory factors expression	^661^
	BET bromodomain inhibitor 2	Not specified	It is a N-Methylpyrrolidone compound which acts as a mimetic of acetyl-lysine and has enhanced affinity as inhibitor of BRD4	^662^
	BET-IN-12	TNBC	This BET-inhibitor is a triazole-containing carboline derivative (2-{8-fluoro-3-[4-(2H3)methyl-1-methyl-1H-1,2,3-triazol-5-yl]-5-[(S)-(oxan-4-yl)(phenyl)methyl]-5H-pyrido[3,2-b]indol-7-yl}propan-2-ol) that has potent anti-tumor activity even at low doses	^663^
	GSK1324726A (I-BET726)	Neuroblastoma	Selective inhibitor of BRD2, BRD3, and BRD4, which downregulates the expression of MYCN and BCL2.	^664^
	SDR-04	Cancer cell lines	It is a 3-methyl-1H-indazole derivative that is selective to BRD4 and mediates anti-proliferative activity by preventing the activation of BRD4 targets like c-MYC	^665^
	BRD4 Inhibitor-19	U266 cancer cells	It is a 3,5-dimethylisoxazole derivative that is a potent inhibitor of BRD4	^666^
	CF53	Leukemia, Breast cancer	Orally active bromodomain and extra-terminal (BET) protein inhibitor	^667^
BET + CBP/p300	NEO2734	NUT midline carcinoma (NMC)	The Dual inhibitor of BET and CBP/p300 bromodomain imparted greater proliferation inhibition and tumor regression	^522^
	XP-524	Pancreatic ductal adenocarcinoma	XP-524 inhibited KRAS/MAPK signaling	^668^
CBP/p300	I-CBP112 hydrochloride	Leukemia	Acts as a competitive inhibitor to the acetyl-lysine protein-protein interaction and prevents self-renewal of acute myeloid leukemia cells.	^431^
	A-485	NSCLC	A spiro-oxazolidinedione which targets and inhibits the HAT domain of CBP/p300 and reduces histone acetylation marks thereby restricts lung cancer cell proliferation through autophagic pathway activation	^669^
	Garcinol	Esophageal cancer	It is a poly-isoprenylated benzophenone derivative from the rind of *Garcinia indica* fruit and is found to inhibit p300 HAT activity by affecting the lysosomal pathway	^670^
	Anacardic acid	Colorectal cancer	It is a natural HAT inhibitor of p300 and PCAF which is extracted from cashew nut shell liquid.	^671^
	Lys-CoA	Melanoma	Selective synthetic inhibitor of p300 HAT activity	^672^
	B026	Leukemia	A potent CBP/p300 small molecule inhibitor that achieved significant dose dependent tumor growth inhibition in MV-4-11 xenograft leukemia mice model	^673^
	YO8197	Prostate cancer	A novel 1-(indolizin-3-yl) ethenone derivative selectively targets bromodomain of CBP/p300 in prostate cancer cell lines and significantly downregulates cMYC and ERG.	^674^
	Nordihydro-guaiaretic acid (NDGA)	HEK293T, HeLa, MEF	It is a natural p300 acetyltransferase activity inhibitor which has been reported to increase lifespan in both flies and mice and induces autophagy.	^675^
	C646	Lung cancer	A selective small molecule inhibitor of p300 has been shown to radiosensitize lung cancer cell lines by inducing mitotic catastrope	^676^
	FT-6876	Breast Cancer	Selective bromodomain inhibitor of CBP/p300 which inhibits acetylation of H3K27Ac at specific promoter sites	^677^
	CCS1477 (Inobrodip)	Prostate cancer, multiple myeloma	Bromodomain inhibitor of CBP/p300	^678,679^
	Melatonin	Breast Cancer	Inhibits p300 activity	^680^
BAP1	iBAP-II	SCLC	Disrupts BAP1/ASXL3/BRD4 epigenetic axisand inhibits small cell lung cancer cell viability and growth in vivo	^681^
CRTC2	Artepillin C	Obesity	Natural compound from propolis, inhibits CREB-CRTC2 axis and reduces lipid levels, enhances insulin sensitivity and decreases fasting glucose levels	^682^
	A57	Obseity	Artepillin C derivative. Higher inhibitory action against CREB-CRTC2 interaction	^438^
PC4	AG-1031	NSCLC	Inhibits ds DNA binding activity of PC4	^438^
DDX5	RX-5902	Renal cell cancer, pancreatic carcinoma, advanced solid tumors, breast cancer	A 1-(3,5-dimethoxyphenyl)-4-[(6-fluoro-2-methoxyquinoxalin-3-yl) aminocarbonyl] piperazine that binds to p-tyr-593 of DDX5 and inhibits its ATPase activity. It is in phase I/II clinical trial. NCT02003092	^683–686^
	Simvastatin	Renal cell carcinoma (RCC)	Inhibits DDX5 and upregulates DUSP5 to inhibit RCC proliferation and metastasis	^687^
TRIM24	Acetyl-lysine mimetic benzimidazolones TRIM24 bromodomain inhibitors	Not specified	TRIM24 bromodomain inhibitor	^688^
MRTFs	CCG-222740	Pancreatic adenocarcinoma	Inhibits RHO/MRTF pathway and modulates inflammatory activity	^689^
BCL9/BCL9L	Carnosic Acid	CRC	Disrupts β-catenin/BCL9 protein–protein interaction	^690^
SET7	Cyproheptadine	Monocytes	In monocytes, cyproheptadine inhibits SET-7 induced persistent activation of malate dehydrogenase and succinate dehydrogenase and thereby, disrupts mitochondrial homeostasis.	^691^
SET7	Cyproheptadine	Breast Cancer	In breast cancer, cyproheptadine destabilizes ERα-mediated gene activation	^692^

Analogous to chemotherapeutic drugs, SMIs often confer resistance by inducing shift in cellular state. For instance, a study by Sun et al.^549^ have reported that TEAD-YAP protein-protein interaction inhibition using TEAD auto-palmitoylation inhibitor MGH-CP1 induces a transient static cell state instead of abrogating the cell population. Contrarily, some inhibitors can perform multiple targeting that can minimize drug resistance; however, it can increase significant risk of toxicity.^550^ Drug efflux is another concern that disables the functionality of SMIs.^551^ Moreover, inability to selectively target the diseased cells and off-target toxicity are the areas of particular concern, leading to the surge for more consistent alternatives.

### Alternate strategies in targeting co-activators

The overreliance on stereotypical idea of antagonistic or agonistic pharmacological perception has limited the reach of small molecule-based strategies, causing substantial stagnation in therapeutic innovation, because of the lack of pursuing some of the best characterized potential target molecules in life-threatening diseases. A promising alternative to this can be the modulation of the disease-causing protein by chemically redirecting them towards the cellular ubiquitin proteasome system for degradation. This approach has been practically implemented via development of proteolysis-targeting chimeras (PROTACs) and molecular glue degraders, the latest discoveries in the field of biomedical research. Although these techniques are still at the bench, rigorous research is being implemented in order to bring them to the bedside.

#### Molecular glue degraders

Molecular glue degraders offer an intriguing targeting strategy by sub-stoichiometrically catalysing the rapid degradation of inaccessible targets. Molecular glues are monovalent molecules of <500 Da that induces interaction between an E3 ubiquitin ligase and the target protein by reshaping the surface of the E3 ligase substrate receptor. As a consequence of this interaction, the target protein degradation takes place^552^ (Fig. 6b). Molecular glue degraders show great potential for treating diseases such as developmental diseases, molecular disorders and also cancer. Brownsey et al.^553^ have explored the application of linkage vector on A-485 conjugated with molecular glue pomalidomide, in targeted degradation of CBP/p300 in myeloma cell line. Using CRISPR/Cas9 knockout screens, a study established a JQ1 based monovalent degrader compound for BRD4 degradation.^554^ Ling et al.^555^ confirmed that the small molecule inhibitor FL118 can act as a molecular glue degrader by interacting with the co-activator DDX5 to promote tyrosine dephosphorylation and subsequent proteasomal degradation without affecting the mRNA levels of the target protein. Moreover, studies have stressed on the importance of molecular glue degraders as a remedy for neurodegenerative diseases,^556^ though their broad-scale implementation requires pervasive research efforts in the fields of molecular biology and medicinal chemistry.

#### PROTAC

Proteolysis-targeting chimeras (PROTACs) are one of the most rapidly growing heterobifunctional targeted protein degradation systems that principally contains two functional ligands, an anchor and a warhead, connected by one chemical linker molecule.^557^ The warhead binds to the protein of interest (POI), while the anchor recruits E3 ubiquitin ligase, thereby hijacking the ubiquitin proteasome system of the cell to degrade the POI^557^ (Fig. 6c). Since its discovery in 2001 by Craig M. Crews, PROTAC has progressed to combat several diseases. Two of the proteolytic targeting chimeras, ARV-110 (NCT03888612) and ARV-471 (NCT04072952) have successfully made it to phase-II clinical trials, ARV-110 for metastatic castration-resistant prostate cancer, and ARV-471 for metastatic ER^+^/HER2^−^ breast cancer.^558^

Concomitantly, scientists have successfully proved the efficacy of PROTACs against the following TFs in the context of cancer: nuclear factor-*κ*B (NF-*κ*B),^559^ androgen receptor (AR),^560^ estrogen receptor (ER),^561^ c-MYC,^562^ p53,^563^ STAT3,^564^ STAT5,^565^ and SMAD3.^566^ In addition to targeting the TFs, PROTACS have also been developed against transcription co-activators with an aim to indirectly regulate TF-associated disease phenotypes. Thomas et al.^567^ provided evidence of JET-209 based PROTAC degrader for CBP/p300 in leukemia. Another CBP/p300 targeting PROTAC named “JQAD1” has also been established in neuroblastoma.^386^ Huang et al.^568^ constructed a biologically inspired PROTAC against TRIM24 by coating the poly lactic-co-glycolic acid (PLGA) nanoparticles containing PROTAC degrader with M2 macrophage membrane (MELT) for atherosclerosis. Lee et al.^569^ developed PROTAC for targeting SRC1 through N-degron pathway in vivo, suggesting the usefulness of N-degron pathway-based degraders of disease-relevant proteins. Otto et al.^570^ designed dBET, a PROTAC against BRD4 for reducing BRD4 mediated c-MYC gene expression in colorectal cancer. Till date, most of the studies relevant to PROTAC development against transcription factors and co-activators have concentrated around cancer. However, in the near future, the advent of this strategy will be intended to target developmental and metabolic disorders.

Like SMIs, there are certain limitations associated with the broad spectrum use of PROTACs in disease therapy. For example, it exhibits poor blood-brain barrier permeability, degradation of off-target proteins, differential expression of E3 ubiquitin ligase in different parts of the body, and poor pharmacokinetics.^569,570^ These facts will necessitate further improvements in PROTACs to increase their efficacy and target specificity.

## Drug repurposing

Repurposing approved drugs is currently a novel approach for disease treatment and is gaining immense popularity, since it may be implemented without facing the impediment imposed by extensive trials and delayed approvals. Strategizing the use of clinically approved drugs not only minimizes the timely and costly endeavours associated with drug development but also provides effective, safer, and cheaper drugs.^571^ Amongst the inhibitors of TF co-activators, there are several FDA-approved drugs currently used for different diseases. Verteporfin, an inhibitor of YAP/TAZ, is an FDA-approved drug for the treatment of age-related macular degeneration, pathologic myopia or presumed ocular histoplasmosis.^572^ Carnosic acid, a component of rosemary extract that is FDA-approved for use as food additives, is an inhibitor of transcriptional co-activators BCL9/9L.^572^ Under inhibitors of CBP/p300 histone acetyl transferases, melatonin is a recognized dietary supplement that falls under FDA’s Dietary Health and Education Act, and cyproheptadine is a clinically approved drug for perennial and seasonal allergic rhinitis, vasomotor rhinitis and allergic conjunctivitis.^572^ Therapeutic repurposing of these drugs for targeting the transcriptional co-activators can thereby be an alternate and effective pharmacological strategy across several pathological conditions.

## Conclusion

Efficient transcriptional signaling mediated by transcription factors is often dependent on the transcriptional co-factors that either physically associate with and/or biochemically modify the genome to reinforce target gene activation or repression.^1,7^ This “soft-wiring” integration of different biological pathways via co-activator action is responsible for modulation of transcriptional activity of several transcription factors across different pathological conditions.^16^ Transcription factors are foundations of the regulatory circuits responsible for selective gene expression.^2,3,6^ Gene expression deregulation is perhaps an inevitable driver of a variety of physiological disorders, including developmental disorders, metabolic disorders, and cancer.^1^ However, modulation of transcription factors that regulate disease-driving genes is one of the most arduous ventures in the field of drug discovery because of its high degree of intrinsic complexity in terms of both structure and interactions.^4,5^ Therefore, we sought to identify their ‘partners-in-crime’, the transcriptional co-activators, and the molecular correspondence underpinning the regulatory circuit of gene regulation. This will aid the development of indirect yet effective targeting strategies against disease-driving transcription factors and will eventually help in better patient prognosis. An up-to-date account on co-activator involvement in diverse disease phenotype and several therapeutic strategies like small molecule inhibitors, molecular glue degrader and PROTACs for targeting the activity of these co-activators have been discussed extensively in this review.

Strikingly, the co-activators can be of immense use to treat diseases where a significant upregulation in their expression has been observed or the primary driver of the disease is not known. Against this backdrop, development of therapeutic strategies to modulate the activity of transcriptional co-activators like PGC-1α that mediates cellular and mitochondrial homeostasis can be a promising therapeutic approach.^208,209^ Angiogenesis is another important regulator of several disease conditions like neurodevelopmental disease and cancer. CRTC family of transcriptional co-activators that have been reported to maintain vascular physiology can be promising therapeutic target.^573^ Hence, a better understanding of the interplay between key cellular compartments, cellular niche and the transcriptional co-activators will help identify alternative therapeutic targets.

However, targeting co-activators may pose certain impediments since they not only play crucial role in disease progression, but also regulate other physiological mechanisms that are necessary for the survival of the organism. For example, CBP/p300 serves as a co-activator for multiple transcription factors like ER,^387^ AR,^574^ NF-*κ*B^575^ amongst others. This addiction of the co-activators for multiple transcription factors poses a challenge for the researchers in the field of drug discovery.^576^ Presently, a multitude of new modifications in the inhibitors are being incorporated to facilitate specific and targeted delivery to the cells of interest. For example, in the context of cancer, delivery of the drugs to the tumor through exosomes,^577^ liposomes^578^ or nanoparticle-mediated delivery systems^579^ may have the potential to achieve desired anti-tumor effects without major risk of off-target toxicity.

Looking towards the future, more co-activators are yet to be discovered, especially in the arena of the cancer stem cells, enabling us to improve our ability to modulate this particular class of regulatory molecules. This review has been an attempt to address an issue that has not been dealt with in a comprehensive manner and hopes to direct attention towards future research that will encompass patient-friendly therapeutic strategies, where drugs will have enhanced benefits and reduced side effects. This will be of considerable potential since utilization of these modulators in combination with conventional chemotherapeutic drugs will overcome the frequently observed phenomenon of cancer recurrence, and additionally treat various developmental and metabolic disorders with elan and success.
